# Targeting resident memory T cell immunity culminates in pulmonary and systemic protection against *Brucella* infection

**DOI:** 10.1371/journal.ppat.1008176

**Published:** 2020-01-17

**Authors:** Hongbin Wang, Carol Hoffman, Xinghong Yang, Beata Clapp, David W. Pascual

**Affiliations:** Department of Infectious Diseases and Immunology, University of Florida, Gainesville, Florida, United States of America; University of São Paulo FMRP/USP, BRAZIL

## Abstract

Brucellosis remains the most common zoonotic disease globally. Currently no vaccines for humans exist, and conventional brucellosis vaccines for livestock fail to confer complete protection; hence, an improved vaccine is needed. Although *Brucella* infections primarily occur following a mucosal exposure, vaccines are administered parenterally. Few studies have considered mucosal vaccinations, or even targeting of tissue-resident memory T (T_RM_) cells. T_RM_ cells protect against viral infections, but less is known of their role in bacterial infections, and even less for brucellosis. Oral prime, nasal boost with a newly developed *Brucella abortus* double mutant (znBAZ) confers nearly complete protection against pulmonary challenge with wild-type (wt) *B*. *abortus* 2308, and its protective efficacy is >2800-fold better than the RB51 vaccine. Vaccination with znBAZ potently stimulated CD8^+^ T cells, whereas mucosal vaccination with RB51 induced mostly CD4^+^ T cells. Subsequent analysis revealed these pulmonary CD44^+^ CD69^+^ CD8^+^ T cells to be either CD103^+^ or CD103^-^ T_RM_ cells, and these sequestered to the lung parenchyma as CXCR3^lo^ and to the airways as CXCR3^hi^. Both CD8^+^ T_RM_ subsets contained single-positive IFN-γ and TNF-α, as well as, polyfunctional cells. IL-17-producing CD8^+^ T_RM_ cells were also induced by znBAZ vaccination, but *in vivo* IL-17 neutralization had no impact upon protection. *In vivo* depletion of CD4^+^ T cells had no impact upon protection in znBAZ-vaccinated mice. In contrast, CD4^+^ T cell depletion reduced RB51’s protective efficacy in spleens and lungs by two- and three-logs, respectively. Although anti-CD8 mAb-treated znBAZ-vaccinated mice showed a significantly reduced pulmonary efficacy, this treatment failed to completely deplete the lung CD8^+^ T cells, leaving the CD103^+^ and CD103^-^ CD8^+^ T_RM_ cell ratios intact. Only znBAZ-vaccinated CD8^-/-^ mice were fully sensitive to pulmonary challenge with virulent wt *B*. *abortus* 2308 since CD8^+^ T_RM_ cells could not be induced. Collectively, these data demonstrate the key role of mucosal vaccination for the generation of CD8^+^ T_RM_ cells in protecting against pulmonary challenge with virulent *B*. *abortus*.

## Author summary

Brucellosis is the most common zoonotic disease worldwide and is transmitted via the consumption of unpasteurized dairy products or exposure to *Brucella*-laden aerosols. In fact, mucosal exposure is the most common route of infection for humans and animals, yet parenteral vaccination of livestock remains the preferred route of immunization. To determine whether development of a mucosal vaccination regimen could effectively generate immunity against pulmonary challenge with virulent *B*. *abortus*, a double-mutant *B*. *abortus* vaccine was administered mucosally, and found to induce CD8^+^ T_RM_ cells. These conferred complete protection against pulmonary infection and prevented systemic brucellae spread even in the absence of immune recirculating CD8^+^ T cells. These data show that mucosal vaccination can stimulate the induction of T_RM_ cells, which should be considered as a more effective means to protect against brucellosis. Furthermore, brucellosis needs to be considered a mucosal pathogen to warrant development of approaches different from conventional methods to protect humans and livestock against this disease.

## Introduction

*Brucella* species are Gram-negative, facultative intracellular bacteria, which are responsible for the most common zoonotic disease worldwide [1,2,3]. Brucellosis in humans is not usually fatal, but it can be debilitating with an undulating fever. Brucellosis can become chronic, leading to lifelong complications including arthritis, osteomyelitis, endocarditis, and meningitis [2,4–7]. Protection against *Brucella* infection requires Th1-type immunity, characterized by the production of IFN-γ and TNF-α. CD4^+^ and CD8^+^ T cells are considered to be the primary source of these protective cytokines during *Brucella* infections [8–10]. The importance of the T cell subsets varies by the route of immunization and the vaccine components [11–18]. Recent reports showed that mucosal exposure to this pathogen stimulates IFN-γ-producing CD8^+^ T cells for protection against brucellosis, not by their cytotoxic function [19–23].

Pioneering work defined two subsets of memory T cells based on the function and migratory properties of these cells [24,25]. Central memory T cells (T_CM_) are found predominantly in secondary lymphoid organs and express lymph node (LN) homing receptors, chemokine receptor CCR7, and the L selectin (CD62L) [24–27]. Lacking CD62L and CCR7 expression, effector memory T (T_EM_) cells migrate among nonlymphoid tissues, LNs, and blood [28–30]. Recent evidence suggests the existence of tissue-resident memory T (T_RM_) cells that are distinct from T_CM_ and T_EM_ cells. T_RM_ cells reside within the epithelium, creating an interface between the host and the environment. They are present in the gastrointestinal (GI) tract, respiratory tract, reproductive tract, and skin [24–27,30]. Noncirculating CD8^+^ T_RM_ cells reside in the mucosal epithelium and keep infections in check by their capacity to immediately recognize and respond to reencountered pathogen [31–38]. Their role in protective immunity at front-line sites of infection is independent of recirculating memory T cells [33,35,39–42]. In addition to CD44 expression, resting T_RM_ cells are characterized by expression of the activation marker CD69 [30,43,44], with the absence or presence of αE integrin (CD103) [27,35]. Moreover, *in vivo* antibody (Ab) labeling studies provide a practical method to discern between tissue-localized cells and recirculating cells in the lungs [45–48]. The identification of T_RM_ cells and their robust protective capacity for tissue-specific infection provides a powerful, new tool for rational vaccine design [37,44,49].

Antigen-specific CD8^+^ T_RM_ cells play a crucial role in controlling pulmonary viral infections [50–53]. The induction of pulmonary CD8^+^ T_RM_ cells has been notably demonstrated subsequent to vaccination or infection with influenza virus [46,48,54–57]. However, the role of CD8^+^ T_RM_ cells following a bacterial infection is less clear since most studies focused on CD4^+^ T_RM_ cells [58, 59]. To address this void and to determine whether T_RM_ cells can be induced for protection against a mucosal challenge with virulent *B*. *abortus*, we conducted oral prime, nasal boost vaccination with a double-mutant *B*. *abortus-lacZ* (znBAZ) strain and measured T_RM_ cell responses. Indeed, znBAZ vaccination, but not conventional livestock RB51 vaccination, enhanced the recruitment of both CXCR3^lo^ CD103^+^ and CXCR3^hi^ CD103^-^CD8^+^ T_RM_ cells into lung parenchyma and airways, respectively. This selective recruitment and/or development of these T_RM_ cells distinguishes the znBAZ from the RB51 immune response. An in-depth analysis was conducted to further characterize these T_RM_ cells, their function, and localization to ultimately understand how these cells potently protect against pulmonary infection with wt *Brucella*. A significant attribute of znBAZ is its ability to stimulate CD8^+^ T_RM_ cells. This contrasts with RB51-induced immunity where CD4^+^ T cells were the primary source of defense, and reaffirms the notion of CD4^+^ T cell-dependence for protection to brucellosis. These studies demonstrate that approaches to stimulate T cell immunity via an alternative mechanism, e.g., CD8^+^ T_RM_ cells, warrant consideration for protection against virulent *Brucella* and other bacterial infections.

## Materials and methods

### *Brucella* strains

The live attenuated *Brucella abortus* mutant, znBAZ, lacks *znuA* and *norD*, and expresses *E*. *coli lacZ*. Development of this strain was previously described [19]. All *B*. *abortus* strains, znBAZ, RB51, and wild-type (wt) *B*. *abortus* strain 2308 were inoculated on Potato Infusion Agar (PIA) plates, and grown for 3 days at 37°C under 5% CO_2_. Brucellae were harvested with sterile phosphate-buffered saline (sPBS), washed twice, and diluted with sPBS for use.

### Ethics statement

All the animal experiments described in the present study were conducted in strict accordance with the recommendations in the Guide for the Care and Use of Laboratory Animals of the National Institutes of Health. All animal studies were conducted under protocols approved by the University of Florida Institutional Animal Care and Use Committee (IACUC # 765). All efforts were made to minimize suffering and ensure the highest ethical and humane standards.

### Mice

Experiments were performed using 6 to 8 week-old female mice. BALB/c and C57BL/6 mice were obtained from Charles River Laboratory (Frederick, MD, USA). CD8-deficient (CD8^-/-^) B6 mice were obtained from Jackson Laboratory (Bar Harbor, ME, USA). All animal experiments performed with live attenuated *Brucella* vaccine strains, znBAZ and RB51, were conducted under biosafety level-2 (BSL-2) containment, and with wt *B*. *abortus* 2308 under BSL-3 containment. Animals were maintained in individually ventilated cages under HEPA-filtered barrier conditions of 12 h of light and 12 h of darkness and provided with food and water ad libitum. All animal care and procedures were in accordance with institutional policies for animal health and well-being and approved by University of Florida IACUC.

### Vaccination

Mice (n = 5-8/group) were orally primed on day 0 with 200 μl of 2.5% sterile sodium bicarbonate in sPBS via a 20-gauge stainless steel feeding tube (Cadence Science, Inc., USA) attached to a 1-ml syringe (BD Becton Dickinson, USA) into mice stomach for 10 mins followed by oral gavage of 200 μl of sPBS, RB51 (1×10^9^ CFUs), or znBAZ (1×10^9^ CFUs). Four weeks after oral prime, mice were nasally dosed with 30 μl (high volume) of sPBS, RB51 (1×10^8^ CFUs), or znBAZ (1×10^9^ CFUs) administered dropwise using a micropipette into the anterior nares under isoflurane anesthesia. Also tested were mice nasally primed and orally boosted. Individual mice lungs and spleens were harvested 42 and 56 days after oral prime. The actual inoculum CFUs was confirmed by serial dilutions of inoculum on PIA plates.

### Vaccine efficacy studies

Mice (n = 5-8/group) were orally primed and nasally boosted with sPBS, RB51, or znBAZ on days 0 and 28 as described above. Then mice were nasally challenged with 5×10^4^ CFUs of wt *B*. *abortus* 2308 on day 56. Two weeks or four weeks after challenge, individually collected spleens and lungs were mechanically homogenized in 1 ml of sterile water with Tissue Lyser (QIAGEN, Germantown, MD). Briefly, ten-fold serial dilutions of homogenates were inoculated on PIA plates to determine the extent of brucellae colonization after 3 to 5 days’ incubation at 37°C under 5% CO_2_. The protective capacity was determined by comparing the CFUs/tissue from RB51- or znBAZ-vaccinated mice to those dosed with sPBS. Extent of splenic inflammation was determined by weighing the spleens. All experiments were repeated two or three times.

### Preparation of single cell suspension and *in vitro* stimulation

Spleens, lower respiratory (mediastinal) lymph nodes (LRLNs), and lungs were collected into 2 ml tubes containing 1 ml of incomplete media (ICM): RPMI-1640 (Caisson Labs, Inc., Smithfield, UT); 10 mM HEPES buffer (Caisson Labs), and 10 mM penicillin/streptomycin (Caisson Labs). Tissues were homogenized (Tissue Lyser; QIAGEN), then filtered through 70 μm disposable cell strainer (Fisherbrand) and washed with ICM at 1500 rpm for 5 mins under 4°C. Splenic red blood cells were lysed by 5 ml of freshly made ammonium-chloride-potassium (ACK) buffer (0.15 M NH_4_Cl, 10 mM KHCO_3_, 0.1 mM Na_2_EDTA) for 5 mins. Lung homogenates in 2 ml of ICM were digested with 20 μg of Liberase TL Research grade (Roche Life Science, Indianapolis, IN) and 50 units of RNase-free DNase I (Promega Corp., Madison, WI), incubated at 37°C under 5% CO_2_, with gentle shaking for 45 mins, and digestion was stopped upon addition of 5 μl of 0.5 M EDTA for 5 mins. Lung cells were treated with 5 ml ACK buffer for 3 mins, and then passed through 70 μm of disposable cell strainer and washed with ICM, and resuspended in Complete Media (CM): ICM plus 10% fetal bovine serum (Atlanta Biologicals, Norcross, GA), 10 mM nonessential amino acids (Caisson Labs), and 10 mM sodium pyruvate (Caisson Labs).

For the studies using separated lung parenchyma and bronchoalveolar lavage fluid (BALF) cells, mouse lungs were perfused twice with 1ml of sPBS, and collected cells from perfusion are designated as BALF cells. The remaining lungs were processed as described above, and designated as parenchyma cells. Collected cells were enumerated using a Cellometer Auto Cell Counter (Nexcelom, Bioscience). Lymphocytes were cultured *in vitro* (2×10^6^ cells/well) in Corning Costar 48-well cell culture plates in 1 ml CM plus stimulators described here. For flow cytometry analysis, one portion of the cells was stimulated overnight with 10^9^ CFUs/ml of heat-killed RB51 (HKRB51) followed by 5 hours of 5 ng/ml phorbol myristate acetate (PMA) (SIGMA-ALDRICH) and 500 ng/ml ionomycin (SIGMA-ALDRICH). Brefeldin A (10 μg/ml; eBioscience, San Diego, CA) was added during the 5 hr stimulation to block cytokine secretion. Stimulated cells were stained with fluorescently labeled monoclonal antibodies (mAbs) to T cell surface antigens and cytokines.

### Cytokine ELISAs

Lung and splenic mononuclear cells were cultured *in vitro* (2×10^6^ cells / well) in 48-well plates in 1 ml CM, and stimulated with 10^9^ CFUs/ml HKRB51 for 84 hours. Supernatants were evaluated for IFN-γ, TNF-α, IL-4, IL-10, and IL-17a by capture ELISA using mAb pairs as previously described [22,23]. Production of cytokines by unstimulated cells was subtracted from all measurements.

### T cell depletion studies

To assess the protective contribution of CD4^+^ and CD8^+^ T cells, BALB/c, B6, and CD8^-/-^ mice were mucosally vaccinated with sPBS, RB51, or znBAZ as described above. Four weeks after the boost, all mice were challenged with wt *B*. *abortus* 2308, and on day 55 (one day before challenge), groups of BALB/c mice were treated intraperitoneal (IP) with 300 μg of rat IgG2b isotype control, anti-CD4 (GK1.5, BioXCell, West Lebanon, NH), or anti-CD8α (2.43, BioXCell) mAbs. Additional Ab treatments were given by IP injection of 200 μg of each mAb or isotype control on days 2, 6, and 10 post-challenge. Two weeks after challenge (day 70), spleens and lungs were individually harvested to determine their T cell profile. Flow cytometry analysis of peripheral blood lymphocytes at day 56 after oral prime, and on the last day of the experiment confirmed the depletion of CD4^+^ and CD8^+^ T cells.

### *In vivo* antibody labeling

To distinguish resident T cells from recirculating T cells in the lungs, *in vivo* Ab labeling of CD4^+^ and CD8^+^ T cells in naive and vaccinated mice was accomplished by IV injection of 10 μg PerCP-Cy5.5-coupled anti-CD4 mAb (clone RM4-5, eBioscience) and PE-Cy7-coupled anti-CD8 mAb (clone 53–5.8, eBioscience). After 10 min recirculating, lungs were perfused twice with sPBS, and BALF cells were harvested. After isolation from the lungs, lymphocytes were stained *ex vivo* with a different, noncompeting mAb clone of anti-CD4 (clone RM4-4, BioLegend) or anti-CD8 (clone 5H10, Thermo Fisher Scientific) mAb in addition to fluorochrome-conjugated mAbs specific for other cell surface markers. Stained cells were analyzed by flow cytometry analysis.

### *In vitro* antibody staining and flow cytometry assay

To determine the types of T cell subsets induced by the various brucellosis vaccines and wt *B*. *abortus* infections, lymphocytes were first washed with sPBS, and then stained with Zombie UV fixable viability kit (BioLegend, San Diego USA) to label dead cells. Cells were labeled with mAbs specific for CD4, CD8α, TCR-β, CD45, CD44, CD62L, CD69, CD103, CD11a, CCR7, CD127, KLRG1, CD19, DX5, and γδ-TCR, and then cells were fixed with IC Fixation Buffer (eBioscience, San Diego USA), permeabilized with Permeabilization Buffer (eBioscience, San Diego USA), and followed by intracellular staining with mAbs specific to IFN-γ, TNF-α, granzyme B, perforin, and T-bet. Fluorescence was acquired on a BD Fortessa flow cytometer with BD FACSDiva software. All the fluorescently conjugated mAbs were purchased from (BioLegend, San Diego USA) or (eBioscience, San Diego USA). All samples were analyzed using FlowJo software (Tree Star, Ashland, OR, USA).

### FTY720 treatment

To establish that immune lung CD8^+^ T_RM_ cells are responsible for protection of the lungs after challenge, groups of BALB/c mice were vaccinated with znBAZ or dosed with PBS as described above, and on day 55 (one day before nasal challenge), half of the znBAZ-vaccinated and PBS-dosed mice were given 0.5mg/kg FTY720 (Santa Cruz Biotech., Dallas USA) in sPBS [60] i.p. and daily administrations continued through day 62; the study was terminated on day 63, one-week post-challenge.

### Immunofluorescence

Whole lungs were perfused with sterile PBS and embedded in OCT (Sakura Finetek), snap-frozen at −26°C, and stored at −80°C until processing. All immunofluorescent staining was performed by Reveal Biosciences, Inc. (San Diego, CA). Frozen lung sections were cut at 5 μm and mounted onto positively charged microscope slides. All primary staining was performed overnight at 4°C, and secondary staining, at room temperature for 60 minutes. Tissue sections were blocked for 30 minutes with Novolink Protein Block (Leica) and then stained with rat IgG2a anti-mouse CD8 mAb (clone 53–6.7; Thermo Fisher) and donkey anti-rat IgG Alexa Fluor 488 (Invitrogen); Armenian hamster anti-mouse CD103 mAb (clone 2E7; Thermo Fisher) and goat anti-hamster IgG Alexa Fluor 647 (Invitrogen); and rabbit polyclonal anti-CD44 Ab (Abcam) and donkey anti-rabbit IgG Alexa Fluor 546 (Invitrogen). DAPI was used as a nuclear counterstain. Whole slide images were generated in fluorescence using a Pannoramic SCAN (3D Histech) and imaged using Case Viewer software (Budapest, Hungary) at 400x power.

### Anti-IL-17 mAb treatment

Groups of 7 wk-old BALB/c mice were orally primed, nasally boosted with sPBS, RB51, or znBAZ, and on day 56 post-primary immunization, all mice were nasally challenged with wt *B*. *abortus* 2308 as described above. Half of each group received IP 250 μg of either of rat IgG1 isotype control (clone TNP6A7, BioXCell) or anti-mouse IL-17 mAb (Clone 17F3, BioXCell) on days -1, day 2, 7, and 12 post-challenge. Tissues were harvested 2 days after the last Ab treatment, and evaluated for extent of brucellae colonization.

### Statistical analysis

One-way ANOVA, or Two-way ANOVA with Tukey’s multiple comparisons test were used to compare groups, depending on data sets and analyzed, and results were discerned to the 95% confidence interval. Statistical significance was determined using GraphPad Prism 7.

## Results

### Mucosal znBAZ vaccination confers superior protection against pulmonary Wt *B*. *abortus* challenge via improved immunogenicity

**Fig 1:**
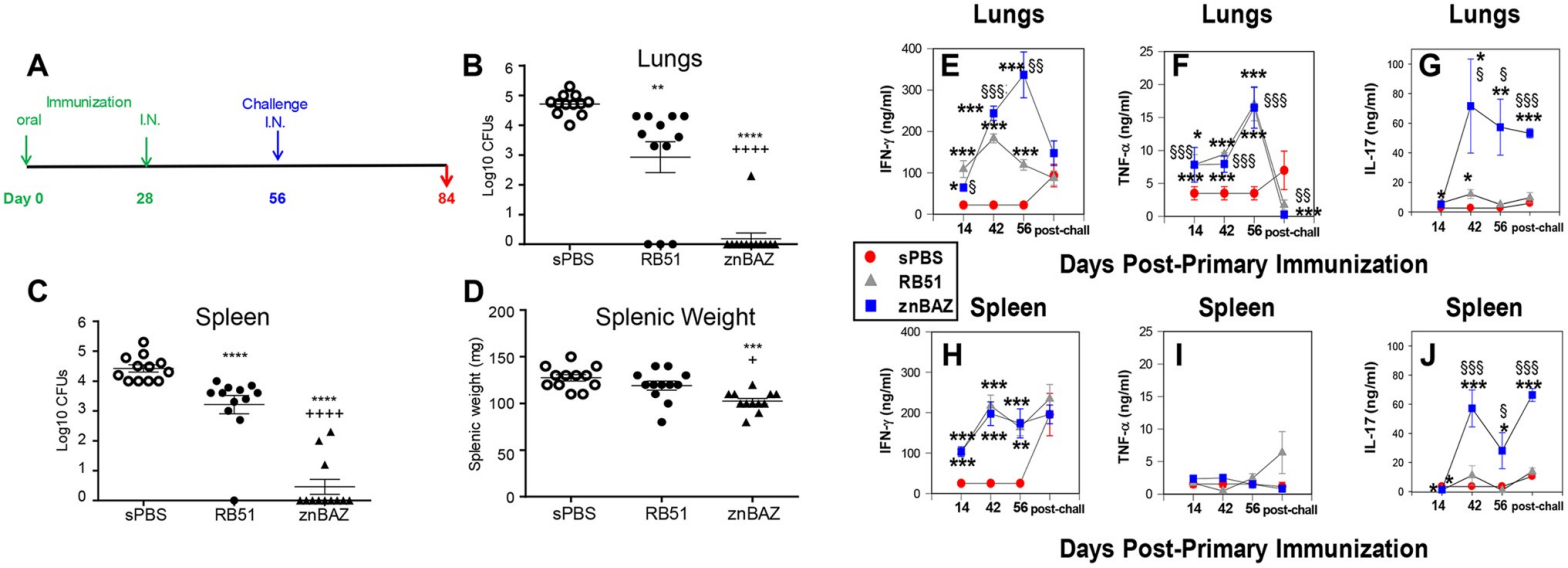
Mucosal znBAZ vaccination and confers superior protection and elicits proinflammatory cytokine production against pulmonary wt *B*. *abortus* infection. (A) Groups of BALB/c mice were orally immunized with 1×10^9^ CFUs of znBAZ, RB51 or sPBS on day 0, and nasally boosted with znBAZ (1×10^9^ CFUs), RB51 (1×10^8^ CFUs), or sPBS on day 28. After 4 weeks, mice were nasally challenged with wild-type (wt) *B*. *abortus* 2308 (5×10^4^ CFUs). To assess vaccine efficacy, 4 weeks post-challenge (n = 12 mice/group), (B) lungs and (C) spleens were assessed for colonization and (D) splenic weights. Data are the mean ± SEM of tissue CFU levels and splenic weights from individual mice. Analysis of variance with Two-way ANOVA Tukey’s multiple comparisons test was performed; ****P<0.0001, ***P<0.001, **P<0.01, *P<0.05, ns indicates not significant versus sPBS-dosed mice; ^++++^P < 0.0001 versus RB51-vaccinated mice. (E-G) Lungs and (H-J) splenic lymphocytes were harvested from separate groups of double-vaccinated mice at 14, 42, 56, and 84 days (4 wks post-challenge [post-chall]) post-primary immunization. Lymphocytes were restimulated with HKRB51 for 3.5 days, and collected supernatants analyzed for (E, H) IFN-γ, (F, I) TNF-α, and (G, J) IL-17a. Data depict n = 8 mice per group, and the mean of two independent experiments. The differences were determined when compared to sPBS-dosed mice: ***P<0.001, **P<0.01, *P<0.05, or compared to RB51-dosed mice ^§§§^P<0.001, ^§§^P<0.01, ^§^P<0.05.

To counter mucosal exposures to *Brucella*, we queried whether mucosal vaccination with znBAZ would prove efficacious against pulmonary challenge with virulent *B*. *abortus*. When administered by the intraperitoneal route, two doses of znBAZ were found to be required for optimal protection [19]. In light of this, groups of BALB/c mice were orally primed on day 0 and nasally boosted on day 28 with znBAZ, RB51, or sPBS. The priming dose for both strains was 1×10^9^ CFUs, and although the booster dose for znBAZ was the same, the booster dose for RB51 had to be reduced 10-fold (1×10^8^ CFUs) since higher doses proved lethal. Booster doses were found to be cleared from the lungs within 3–4 wks. Four wks after the booster dose, mice were nasally challenged with wt *B*. *abortus* 2308 (5×10^4^ CFUs) (Fig 1A). Four weeks post-challenge, their lungs (Fig 1B) and spleens (Fig 1C) were examined to measure the extent of colonization by *B*. *abortus* 2308, and to determine splenic weights (Fig 1D). RB51 vaccination reduced wt brucellae colonization by 15.8- and 63-fold in the spleens and lungs, respectively, when compared to sPBS-dosed mice. znBAZ vaccination conferred nearly sterile protection against pulmonary wt *B*. *abortus* challenge with 75% and 92% of znBAZ-immunized mice showed no detectable CFUs in spleens and lungs, respectively. znBAZ vaccination conferred significantly (P<0.0001) better efficacy by ~2800-fold than was seen in RB51-vaccinated mice (Fig 1B and 1C). In addition, mucosal znBAZ vaccination significantly attenuated splenic inflammation following wt pulmonary challenge relative to sPBS- (P<0.001) or RB51-vaccinated mice (P<0.05) (Fig 1D). The sequence of nasal prime, oral boost versus oral prime, nasal boost did not significantly impact znBAZ’s efficacy against pulmonary challenge with wt *B*. *abortus* 2308 (S1 Fig).

To gain insight into znBAZ’s immunogenicity as a result of mucosal vaccination, lymphocytes isolated at days 14, 42, 56, and post-challenge from lungs and spleens were evaluated for soluble cytokine production subsequent in vitro restimulation with HKRB51 (Fig 1E–1J). Both IFN-γ (Fig 1E) and TNF-α (Fig 1F) peaked at day 56 post-primary immunization with znBAZ, while IL-17 peaked and sustained levels by day 42 post-primary immunization (Fig 1G). IFN-γ and TNF-α production by lung lymphocytes peaked by day 42 and 56, respectively, in RB51-vaccinated mice, and these cytokine levels were significantly greater than in sPBS-dosed mice (P < 0.01). IFN-γ produced by lung lymphocytes from znBAZ-vaccinated mice exceeded levels produced by RB51-vaccinated mice by 1.4- and 3-fold on days 42 and 56, respectively. Four weeks post-challenge, IFN-γ and TNF-α levels produced by lung lymphocytes from znBAZ- and RB51-vaccinated groups were both significantly (P < 0.05) expended from their peak levels (Fig 1E and 1F), signifying their relevance for protection. Splenic IFN-γ levels remained unchanged for both vaccinated group pre- and post-challenge (Fig 1H). No significant differences in splenic TNF-α production were observed at any time points measured for sPBS-, znBAZ-, and RB51-vaccinated mice (Fig 1I). On day 42, IL-17 production by lung lymphocytes was significantly elevated (P < 0.05) 5.9-fold in znBAZ- versus RB51-vaccinated mice, and only then was IL-17 from RB51-vaccinated mice significantly more than that seen in sPBS-dosed mice (Fig 1G). Sustaining elevated levels pre- and post-challenge, lung IL-17 from znBAZ-vaccinated mice was 11- and 5.5-fold greater than IL-17 produced by RB51-vaccinated mice on day 56 and post-challenge, respectively. IL-17 levels from restimulated splenic lymphocytes from znBAZ-vaccinated showed sustained elevated production pre- and post-challenge while those from RB51-vaccinated or sPBS-dosed mice showed minimal production (Fig 1J). Lung and splenic IL-17 did not appear to be expended as a consequence of challenge (Fig 1G and 1J). Collectively, these data demonstrate that oral prime, nasal boost with znBAZ is potently effective in protecting against pulmonary challenge with virulent *B*. *abortus* 2308, representing a significant improvement in efficacy versus RB51 vaccine.

### Mucosal znBAZ induces robust lung CD8^+^ T cell responses

**Fig 2:**
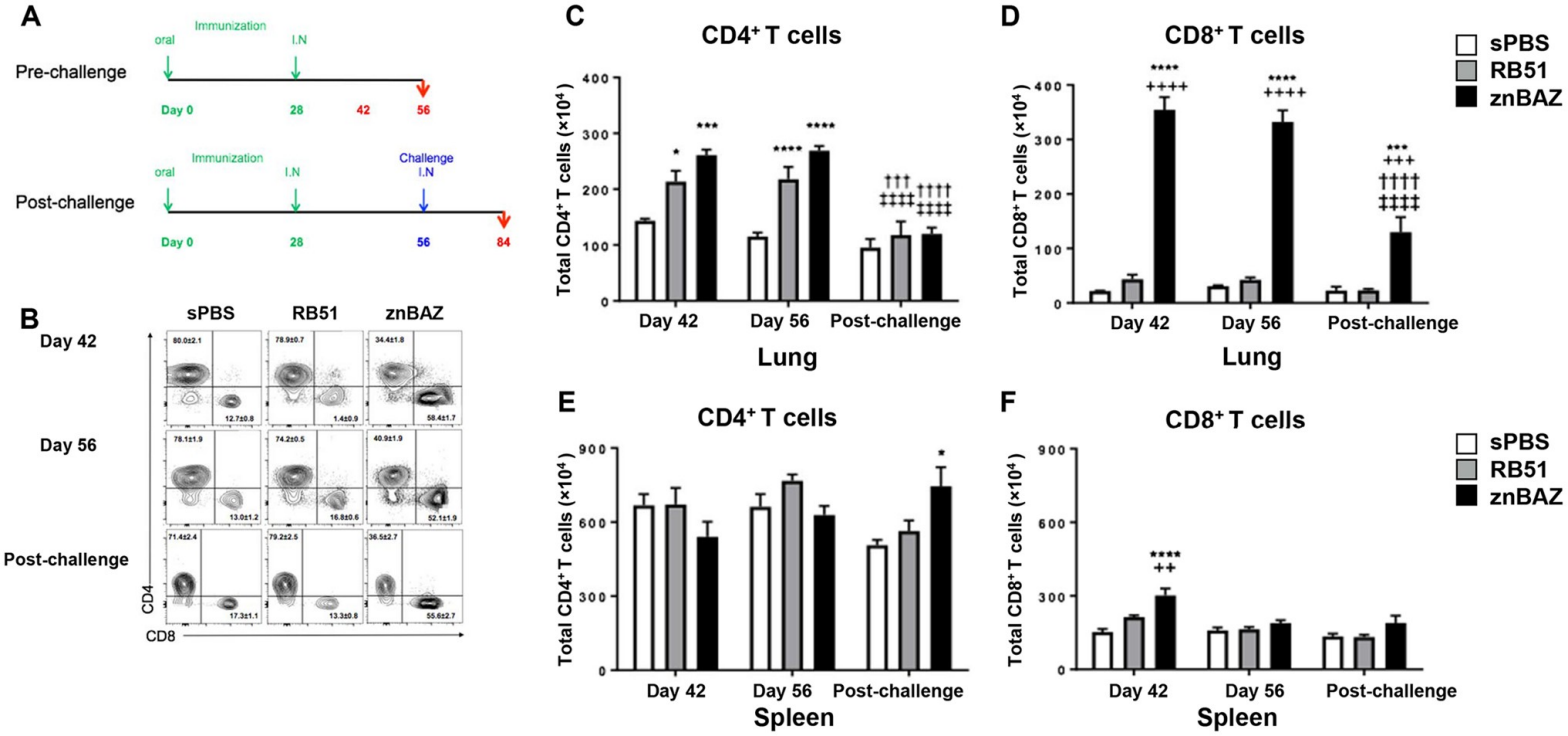
Mucosal znBAZ enhances the recruitment of *Brucella*-specific CD8^+^ T cells into the lungs. (A) BALB/c mice were orally primed and nasally boosted as described. On days 42 and 56, lymphoid tissues were isolated to evaluate their T cell content by FACS analysis. Additional groups of mice, subjected to the same immunization regimen, were challenged with wt *B*. *abortus* 2308 (5×10^4^ CFUs) on day 56. Four weeks post-challenge, lymphoid tissues were isolated to evaluate the T cell infiltration. (B) Flow cytometry of CD4^+^ and CD8^+^ expression from gated TCR-β^+^ cells in the lungs at 42, 56, and 84 days after oral prime. Total numbers of (C, E) CD4^+^ and (D, F) CD8^+^ T cells in the (C, D) lungs and in the (E, F) spleen at various time points. The data depict n = 12 mice per group from two independent experiments. The differences were determined when compared to sPBS-dosed mice (****P<0.0001, ***P<0.001, **P<0.01, *P<0.05), or compared to RB51-vaccinated mice (^++++^P<0.0001, ^+++^P<0.001, ^++^P<0.01, ^+^P<0.05). The difference among the time points were determined when compared to day 42 (^††††^P<0.0001, ^†††^P<0.001, ^††^P<0.01, ^†^P<0.05), or compared to day 56 (^‡‡‡‡^ P<0.0001, ^‡‡‡^P<0.001, ^‡‡^P<0.01, ^‡^P<0.05). Analysis of variance with Two-way ANOVA Tukey’s multiple comparisons test was performed.

To inquire about the mechanisms responsible for this exquisite protection, flow cytometric analysis was performed to determine the types of T cell subsets induced following znBAZ vaccination (Fig 2A). From vaccinated mice as described above with sPBS, RB51, and znBAZ, lung, LRLN, and splenic lymphocytes were evaluated for their immune status at 2 wks after boost (day 42), and on the day slated for challenge (day 56). Additional groups of similarly vaccinated mice were nasally challenged with wt *Brucella* 2308 on day 56, and 4 weeks post-challenge, tissues were also evaluated to measure changes in T cell subsets. Gated on TCR-β^+^ cells, the relative proportions of CD4^+^ and CD8^+^ T cells were measured as a percentage (Fig 2B) and as total cell numbers (Fig 2C and 2D). The results show that the percentage of CD4^+^ T cells is not obviously changed by RB51 vaccination compared to sPBS-dosed mice, but CD4^+^ T cells are nearly reduced in half by znBAZ-vaccinated mice before and after challenge (Fig 2B). Although the total CD4^+^ T cells numbers remained similar between RB51- and znBAZ- vaccinated mice, both znBAZ (P<0.001) and RB51 (P<0.05) show significant increases compared to sPBS-dosed mice. These vaccine-induced increases in CD4^+^ T cells are expended following challenge, resembling levels present in sPBS-dosed mice (Fig 2C).

A stark contrast in CD8^+^ T cell levels is observed the for znBAZ-vaccinated mice evident by the 4- to 5-fold more CD8^+^ T cells present in the lungs than in sPBS-dosed and RB51-vaccinated mice (Fig 2B top and middle row). Notably, this CD8^+^ T cell influx is preserved after nasal challenge with wt *B*. *abortus* (Fig 2B bottom row). Likewise, the total number of CD8^+^ T cells induced in znBAZ-vaccinated mice increases 8-fold relative to RB51-vaccinated mice, and 10- to 16-fold relative to sPBS-dosed mice at days 42 and 56 (Fig 2D). Following wt challenge, the elevated CD8^+^ T cell levels by znBAZ-vaccinated mice remains 6-fold greater than RB51-vaccinated and sPBS-dosed groups, though the levels reduce significantly (P<0.0001) relative to pre-challenge levels (Fig 2D). No obvious changes in the CD8^+^ T cells numbers by the RB51-vaccinated mice relative to sPBS-dosed group (Fig 2B and 2D). Examination of LRLNs and spleens on day 42 reveals that total CD8^+^ T cells increase in the LRLNs following znBAZ vaccination (S2 Fig), in contrast to the slight increase (P<0.0001) in the spleen, which was slightly more (P<0.01) than in RB51-vaccinated mice (Fig 2F). znBAZ vaccination increased (P<0.05) splenic CD4^+^ T cells relative to sPBS-dosed group only after wt challenge with no detectable changes prior to challenge (Fig 2E). RB51 vaccination does not alter splenic CD4^+^ T cell levels before or after wt challenge (Fig 2E). Following mucosal vaccination, znBAZ and RB51 increase pulmonary CD4^+^ T cell recruitment, but this enhancement is not preserved subsequent to wt challenge. Notably, only znBAZ vaccination enhances pulmonary CD8^+^ T cell responses and high levels are maintained post-challenge. These results show that the protective mechanism induced by mucosal znBAZ is different from than the one used by RB51 vaccination.

### Mucosal znBAZ vaccination significantly enhances the IFN-γ- and TNF-α-producing CD4^+^ and CD8^+^ T cells

**Fig 3:**
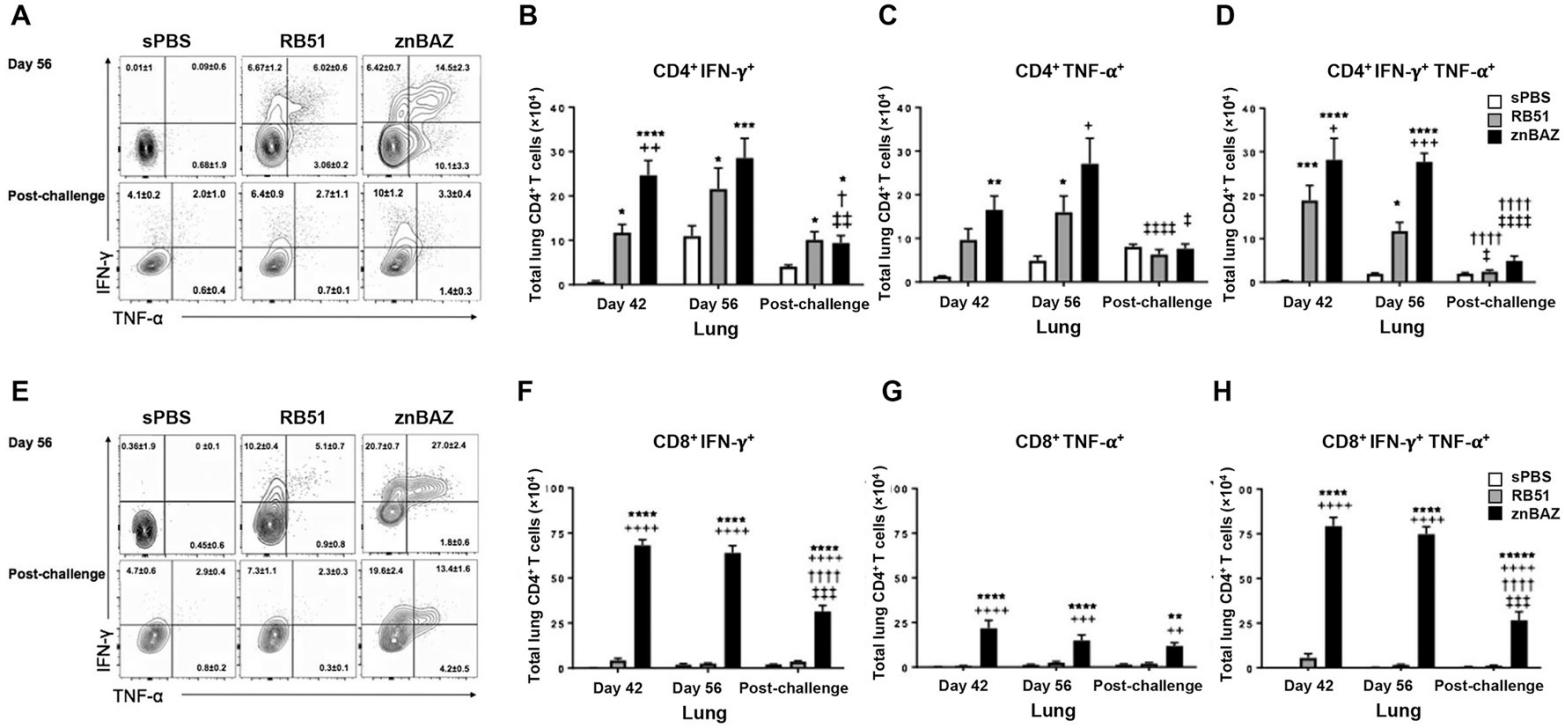
Mucosal znBAZ enhances polyfunctional T cell responses. BALB/c mice were primed and boosted with sPBS, RB51, and znBAZ as described in (Fig 1A). Mice were analyzed for the expression of proinflammatory cytokines pre- and post-wt *B*. *abortus* 2308 challenge. (A) Flow cytometry of IFN-γ and TNF-α expression by CD4^+^ T cells in the lungs on day 56. (B-D) total numbers of IFN-γ- and TNF-α-producing CD4^+^ T cells are depicted for days 42, 56, and 84 post-primary immunization. (E) Flow cytometry of IFN-γ and TNF-α expression on CD8^+^ T cells in the lungs on day 56. (F-H) Total numbers of IFN-γ- and TNF-α-producing CD8^+^ T cells are depicted for the same time points described above. The data depict n = 12 mice per group from two independent experiments. The differences were determined when compared to sPBS-dosed mice (****P<0.0001, ***P<0.001, **P<0.01, *P<0.05), or compared to RB51-vaccinated mice (^++++^P<0.0001, ^+++^P<0.001, ^++^P<0.01, ^+^P<0.05). The differences between time points were determined when compared to day 42 (^††††^P<0.0001, ^†††^P<0.001, ^††^P<0.01, ^†^P<0.05), or compared to day 56 (^‡‡‡‡^ P<0.0001, ^‡‡‡^P<0.001, ^‡‡^P<0.01, ^‡^P<0.05). Analysis of variance with Two-way ANOVA Tukey’s multiple comparisons test was performed.

To investigate the cell source of proinflammatory cytokines induced subsequent to znBAZ vaccination, lung lymphocytes were assessed for IFN-γ and TNF-α production by flow cytometry. The percentage of IFN-γ^+^ CD4^+^ T cells increases from 0.01% in sPBS-to more than 6% in RB51- and znBAZ-vaccinated mice at day 56. A similar increase is observed in IFN-γ^+^ CD4^+^ T cells after wt challenge (Fig 3A). The total IFN-γ^+^CD4^+^ T cells are significantly (P<0.05) elevated by RB51 before (by 12-fold) and after (by 6-fold) wt challenge (Fig 3B). For znBAZ-vaccinated mice, the level of IFN-γ^+^CD4^+^ T cells is 13-fold greater (P<0.01) than in RB51-vaccinated mice on day 42. Compared to sPBS-dosed mice, znBAZ immunization significantly enhances IFN-γ^+^CD4^+^ T cells 24-fold (P<0.0001) at day 42, 19-fold (P<0.001) at day 56, and 5.4-fold (P<0.05) post-challenge. The number of IFN-γ^+^CD4^+^ T cells in the pre-challenge groups is 15- to 19-fold more (P<0.05) than that in the same groups post-challenge (Fig 3B). The proportion of TNF-α^+^ CD4^+^ T cells increases from 0.68% in sPBS- to 3.06% in RB51- or 10.1% in znBAZ-vaccinated mice, while no obvious changes are observed among sPBS and vaccine groups post-challenge (Fig 3A). The number of TNF-α^+^ CD4^+^ T cells is elevated by RB51 immunization, but only at day 56, and less than those in znBAZ-vaccinated mice (Fig 3C). znBAZ vaccination increases the number of TNF-α^+^CD4^+^ T cells by 15- to 22-fold (P<0.01) at all time points before challenge, but their levels are significantly (P<0.0001) abated by wt challenge (Fig 3C). The proportion of polyfunctional IFN-γ^+^ TNF-α^+^ CD4^+^ T cells increased in both RB51- and znBAZ-vaccinated mice at day 56, but this elevation is not maintained after wt challenge (Fig 3A).

The number of lung polyfunctional IFN-γ^+^TNF-α^+^ CD4^+^ T cells increases significantly (P<0.05) more than sPBS-dosed mice to 10- to 18-fold subsequent to RB51 vaccination, and diminishes after wt challenge (Fig 3D). znBAZ vaccination increases the production of polyfunctional IFN-γ^+^TNF-α^+^ CD4^+^ T cells relative to sPBS- (P<0.0001) and RB51- (P<0.05) vaccinated mice. The enhancement is maintained longer, but is mostly consumed by wt challenge (Fig 3D). Likewise, splenic CD4^+^ T cells expressing IFN-γ^+^, TNF-α^+^, or both following RB51- or znBAZ-vaccination are induced, but are consumed by wt challenge (S3A Fig). These data show that both znBAZ and RB51 can enhance the recruitment of CD4^+^ T cells producing IFN-γ, TNF-α, or both, but pulmonary wt *B*. *abortus* 2308 challenge diminishes this enhancement of the cytokine-producing CD4^+^ T cells.

Following znBAZ immunization, CD8^+^ T cells produce notably greater proinflammatory cytokines than those by CD4^+^ T cells. The percentage of IFN-γ^+^CD8^+^ T cells by sPBS-dosed mice remains less than 1%, while znBAZ immunization increases the expression to 20%, which is twice more than those from RB51-vaccinated mice before and after wt challenge (Fig 3E). The number of IFN-γ^+^ CD8^+^ T cells by RB51-immunization is not significantly increased relative to sPBS-dose mice. In contrast, znBAZ immunization dramatically expands the number of IFN-γ^+^ CD8^+^ T cells by 17-fold more than RB51-immunized mice. This is significantly (P<0.0001) more than from sPBS-dosed mice at days 42, 56, and after wt challenge (Fig 3F), though the level of IFN-γ^+^ CD8^+^ T cells is significantly (P<0.0001) reduced after challenge (Fig 3F). Although the proportion of TNF-α^+^ CD8^+^ T cells does not change as a consequence of vaccination or wt challenge (Fig 3E), the total number of TNF-α^+^CD8^+^ T cells following znBAZ vaccination does significantly increase both before (P<0.0001) and after (P<0.01) wt challenge (Fig 3G). However, RB51 vaccination does not alter the number of TNF-α^+^CD8^+^ T cells at any time point (Fig 3G). Notably, the level of polyfunctional IFN-γ^+^TNF-α^+^CD8^+^ T cells is greater than IFN-γ^+^ CD8^+^ T cell levels following znBAZ immunization. The percentage of polyfunctional CD8^+^ T cells increased from 0% in naïve mice to 5.08% in RB51-, and 27% in znBAZ-vaccinated mice (Fig 3E top row). The proportion of the polyfunctional CD8^+^ T cells reduces by half after wt challenge for each vaccine (Fig 3E bottom row). RB51 immunization did not alter the number of the polyfunctional CD8^+^ T cells at any time point (Fig 3H). The total number of polyfunctional CD8^+^ T cells following znBAZ vaccination increases 14- and 46-fold relative to RB51-vaccinated mice on days 42 and 56, respectively (Fig 3H). The enhanced number of the polyfunctional CD8^+^ T cells significantly (P<0.001) decreases upon wt challenge, but still maintains a level 24-fold more than seen in RB51-vaccinated mice (Fig 3H). Splenic IFN-γ^+^, TNF-α^+^, and polyfunctional CD8^+^ T cells increase following znBAZ vaccination, but not after wt challenge. The number of the splenic cytokine-producing CD8^+^ T cells is notably less than those in the lungs (S3B Fig). Collectively, these results demonstrate that mucosal znBAZ vaccination potently induces the stimulation of polyfunctional, as well as, IFN-γ^+^ or TNF-α^+^ CD8^+^ T cells, all of which are maintained after vaccination and following pulmonary challenge with wt *B*. *abortus* 2308. In contrast, RB51 vaccination by the same route stimulates mostly CD4^+^ T cells without altering the CD8^+^ T cells, with the profile resembling that of naïve animals.

### CD8^+^ T cells are essential for znBAZ-mediated protection against pulmonary challenge with virulent *B*. *abortus* in contrast to RB51’s reliance on CD4^+^ T cells

**Fig 4:**
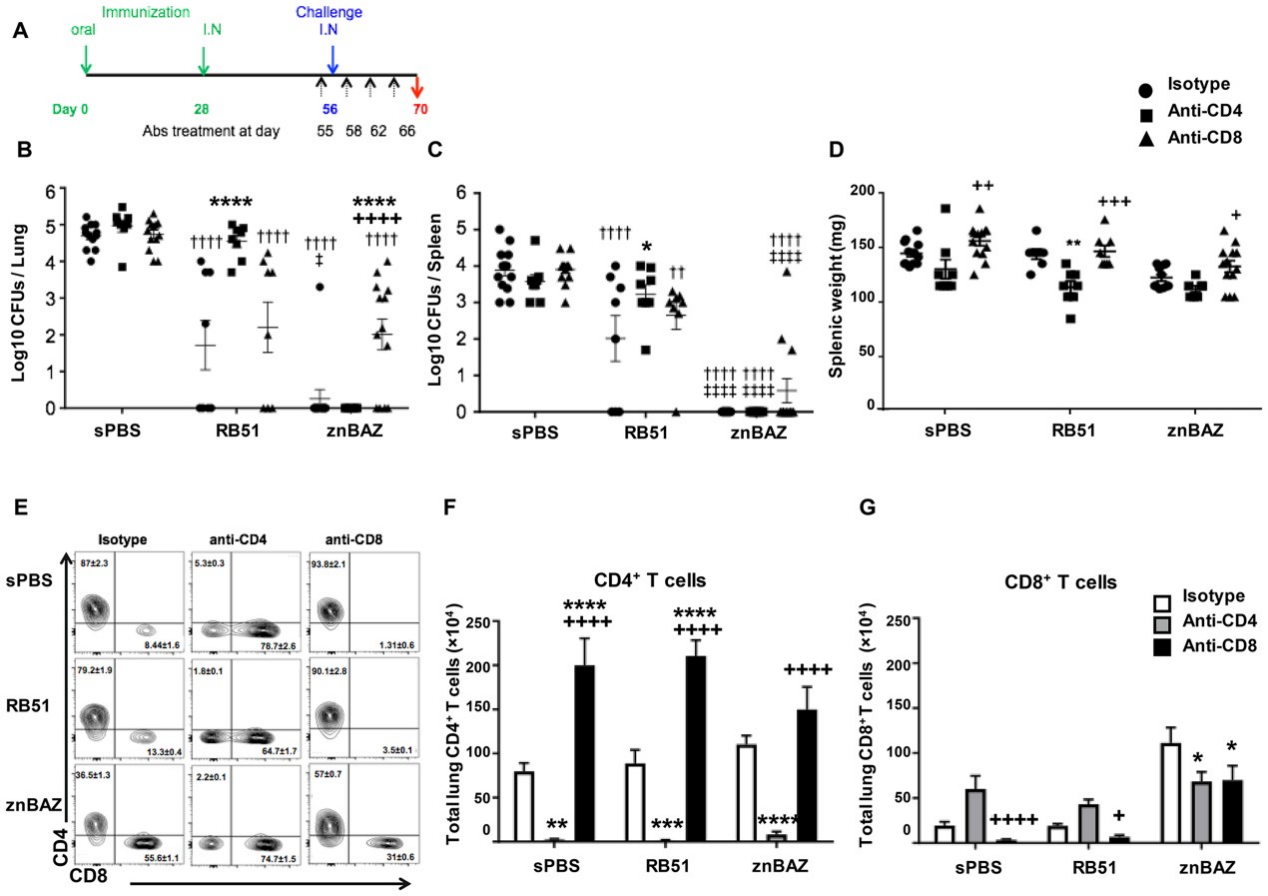
CD8^+^ T cells are critical for znBAZ-mediated protection against pulmonary wt *B*. *abortus* 2308 challenge. BALB/c mice were orally primed and nasally boosted with sPBS, RB51, and znBAZ as described in A. On day 56, all mice were challenged with virulent *B*. *abortus* 2308, and on days 55 (one day before challenge), 57, 62, and 66, mice were treated (IP) with isotype control, anti-CD4, or anti-CD8α mAb. On day 70 (2 weeks after challenge), (B) lungs and (C) spleens were harvested to measure the extent of brucellae colonization, as well as determine their (D) splenic weights and (E-G) T cell profiles. (E) Flow cytometry analysis was performed gating on TCR-β^+^ cells for CD4^+^ and CD8^+^ expression on lung lymphocytes isolated post-challenge following isotype, anti-CD4, or anti-CD8α mAb treatment for each vaccination group. Total numbers of (F) CD4^+^ and (G) CD8^+^ T cells in the lungs are depicted. The data depict n = 12 mice per group combined from three independent experiments. The differences were determined when compared to isotype Ab-dosed mice (****P<0.0001, ***P<0.001, **P<0.01, *P<0.05), or compared to anti-CD4 mAb-treated mice (^++++^P<0.0001, ^+++^P<0.001, ^++^P<0.01, ^+^P<0.05). The differences amongst immunization groups were determined when compared to sPBS group (^††††^P<0.0001, ^†††^P<0.001, ^††^P<0.01, ^†^P<0.05), or compared to RB51 group (^‡‡‡‡^ P<0.0001, ^‡‡‡^P<0.001, ^‡‡^P<0.01, ^‡^P<0.05). Analysis of variance with Two-way ANOVA Tukey’s multiple comparisons test was performed.

Although prominently enhanced, it is unclear whether the znBAZ-induced CD8^+^ T cells are solely responsible for protection or if CD4^+^ T cells are also required. To determine which T cells are responsible for the protective potency induced by mucosal znBAZ, BALB/c mice were vaccinated with znBAZ, RB51, or sPBS as described above, and then subjected to T cell depletion at the time of challenge. To deplete T cell subsets, mice were treated with mAbs specific for CD4^+^ or CD8^+^ T cells or isotype control one day before challenge, and depletions were maintained during the challenge phase (Fig 4A). CD4^+^ and CD8^+^ T cell depletions were confirmed by their absence in peripheral blood. Pulmonary challenge with virulent *B*. *abortus* 2308 was done as described above at four weeks after boost. Two weeks after challenge (day 70), individual spleens and lungs were harvested to measure extent of wt brucellae colonization (Fig 4B and 4D) and induced T cell phenotypes (Fig 4E–4G). Anti-CD4 mAb treatment diminished the protective efficacy of RB51-vaccinated mice by 3000-fold (P<0.0001) in the lungs (Fig 4B) and 1300-fold (P<0.05) in the spleen (Fig 4C). In contrast, protection conferred by RB51 remained unaffected by anti-CD8 mAb treatment showing no change in wt brucellae colonization of the lungs or spleen (Fig 4B and 4C). These data show that CD4^+^ T cells, not CD8^+^ T cells, are critical for RB51-mediated protection against pulmonary infection with wt *B*. *abortus*. In contrast, anti-CD4 depletion had no impact upon protection in mice mucosally vaccinated with znBAZ, since efficacy was unchanged from mice treated with isotype control as evidenced by the lack of wt brucellae in both the lungs (Fig 4B) and spleen (Fig 4C). The potent protection normally achieved for the lungs was significantly (P<0.0001) reduced upon anti-CD8 mAb treatment, yet some protection was retained as evidenced by only a 500-fold reduction in wt brucellae colonization (Fig 4B). These results show that the superior protection conferred by znBAZ immunization is not dependent on CD4^+^ T cells, rather on CD8^+^ T cells. Mice without CD4^+^ T cells from all treatment groups exhibit decreased (no significance to P<0.01) splenic weights when compared to isotype-treated control mice, in contrast to anti-CD8-treated mice showing signficantly (P<0.05) increased splenic weights (Fig 4D). These data suggest that CD4^+^ T cells may be the main contributor to splenic inflammation. Nevertheless, CD8^+^ T cells are essential for znBAZ–mediated protection against pulmonary infection with virulent *B*. *abortus* 2308.

Results from the T cell depletion studies show that protection by znBAZ is CD8 T cell-dependent while protection by RB51 is CD4 T cell-dependent. CD4^+^ T cells are the dominant population in RB51-vaccinated mice, accounting for >80% of total TCR-β^+^ cells. In contrast, the proportion of CD4^+^ T cells by znBAZ-vaccinated mice is 36.5% (Fig 4E left column). Anti-CD4 mAb treatment reduces the proportion of CD4^+^ T cells by more than 95% in each vaccination group (Fig 4E middle column). Despite the effectiveness of anti-CD4 mAb treatment in reducing the number of lung CD4^+^ T cells in all treatment groups by 80- to 110-fold (Fig 4F), anti-CD8 mAb treatment proves ineffective in depleting CD8^+^ T cells recruited into the lungs subsequent to mucosal znBAZ vaccination (Fig 4E and 4G). Anti-CD8 mAb treatment effectively reduces the proportion (Fig 4E) and total cell numbers (Fig 4G) of CD8^+^ T cells in sPBS- and RB51-vaccinated mice. Yet, more than half of the lung CD8^+^ T cells in znBAZ-vaccinated mice remains after anti-CD8 mAb treatment (Fig 4E right column and Fig 4G). Such resistance to mAb penetration is not evident in the spleen since both CD4^+^ and CD8^+^ T cells are susceptible to mAb depletions (S4 Fig). In summary, these data show that CD4^+^ T cells are critical for RB51-mediated protection against pulmonary infection with wt *B*. *abortus*, in contrast to CD8^+^ T cells being essential for znBAZ-mediated protection. In znBAZ-vaccinated mice, the retained lung CD8^+^ T cells are resistant to mAb depletion, and sufficient to confer pulmonary protection, even in the absence of recirculating CD8^+^ T cells.

### Mucosal vaccination induces recruitment of recirculating memory CD69^+^ CD4^+^ T cells into the lungs

**Fig 5:**
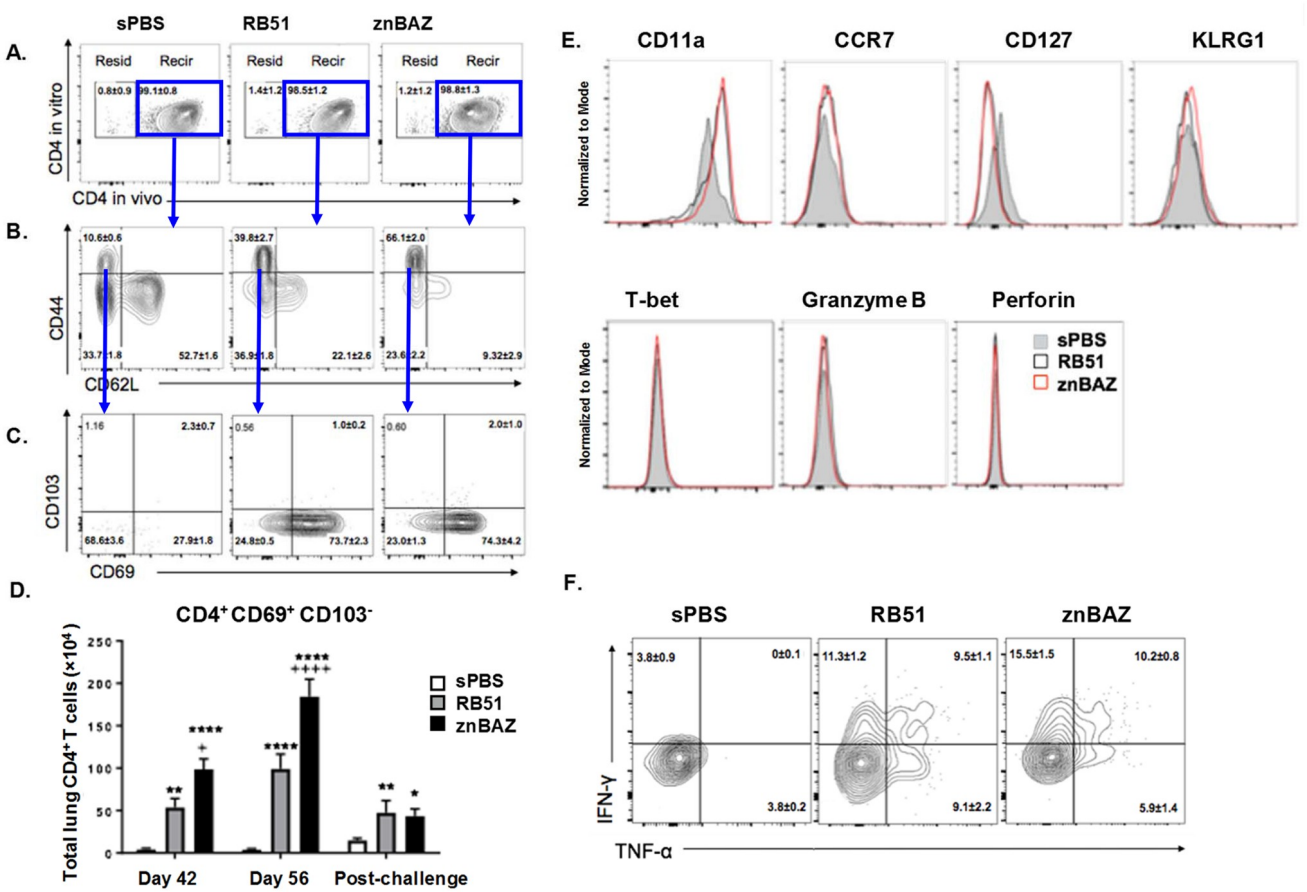
Mucosal znBAZ vaccination enhances the influx of recirculating memory CD44^hi^ CD69^+^ CD4^+^ T cells into the lungs. BALB/c mice were primed and boosted with sPBS, RB51, and znBAZ as described in (Fig 1A). Pre- or post-wt *B*. *abortus* 2308 challenge, mice were analyzed for the expression of resident (Resid) and recirculating (Recir) memory CD4^+^ T cell subsets on days 42 and 56 (pre-challenge), as well as on day 84 (post-challenge). (A) Flow cytometric analysis of lung CD4^+^ T cells in vivo labeled on day 56. (B) FACS plots show the percentage of cells expressing CD62L and CD44, and (C) CD69 and CD103. (D) Total numbers of CD103^-^ CD69^+^ memory CD4^+^ T cells from the lungs at various time points are depicted. The data depict n = 12 mice per group showing the mean ± SEM of three independent experiments. The differences were determined when compared to sPBS-dosed mice (****P<0.0001, ***P<0.001, **P<0.01, *P<0.05), or compared to RB51-vaccinated mice (^++++^P<0.0001, ^+++^P<0.001, ^++^P<0.01, ^+^P<0.05). The difference among time points were determined when compared to day 42 (^††††^P<0.0001, ^†††^P<0.001, ^††^P<0.01, ^†^P<0.05), or compared to day 56 (^‡‡‡‡^ P<0.0001, ^‡‡‡^P<0.001, ^‡‡^P<0.01, ^‡^P<0.05). Analysis of variance with Two-way ANOVA Tukey’s multiple comparisons test was performed. (E) Expression of CD11a, CCR7, CD127, KLRG1, T-bet, granzyme B, and perforin by memory CD103^-^ CD69^+^ CD4^+^ T cells from the lungs on day 56. (F) IFN-γ and TNF-α production by memory CD103^-^ CD69^+^ CD4^+^ T cells from the lungs on day 56.

Inquiring into the composition of the lung-infiltrating T cells following znBAZ vaccination, we examined the phenotype of the induced T cells resembling T_CM_ (CD44^hi^ CD62L^hi^ CD69^lo^), T_EM_ (CD44^hi^ CD62L^lo^ CD69^lo^), or T_RM_ (CD44^hi^ CD62L^lo^ CD69^hi^ CD103^+^ or CD103^-^) cells. An *in vivo* Ab labeling approach [45,46] was used to distinguish tissue resident from circulating T cells, fluorescently-conjugated anti-CD4 mAb was IV administered in order to *in vivo* label the circulating CD4^+^ T cells. This is an effective means to label circulating CD4^+^ T cells leaving tissue-bound cells refractive to labeling [45,46]. Vaccinated mice (sPBS, RB51, and znBAZ) at days 42 and 56 (pre-challenge) as well as vaccinated and challenged mice on day 84 were analyzed for memory CD4^+^ T cell subsets as well as (post-challenge). Gating on TCR-β^+^ cells, ~99% of CD4^+^ T cells were labeled by both *in vivo* and *in vitro* anti-CD4 mAbs identifying them as recirculating cells (Fig 5A–5C). In contrast, only 1% of CD4^+^ T cells were not *in vivo* labeled, and stained only with the *in vitro* anti-CD4 mAb, implicating these are the resident T cells (Fig 5A). To distinguish from memory T cells, gating of the recirculating CD4^+^ T cells was accomplished using CD44 and CD62L, and data indicated that the lung CD4^+^ T cells from sPBS-dosed mice exhibited predominantly (>52.7%) a naive phenotype expressing CD44^lo^CD62L^hi^, and only a small portion (~10%) expressed memory CD44^hi^ T cell phenotype (Fig 5B). Following RB51 or znBAZ vaccination, the naïve CD4^+^ T cell fractions were reduced to 22.1% in the RB51- and 9.32% in the znBAZ-vaccinated mice. None of these CD4^+^ T cells expressed a T_CM_ phenotype (CD44^hi^CD62L^hi^) (Fig 5B). Rather, ~40% and 66% of the recirculating CD4^+^ T cells from RB51- and znBAZ-vaccinated mice exhibited a CD44^hi^CD62L^lo^ phenotype, respectively, resembling an T_EM_ or other memory recirculating CD4^+^ T cells (Fig 5B). To discern the subsets within the recirculating memory CD44^hi^CD62L^lo^ CD4^+^ T cells, further examination for the expression of CD69 and CD103 revealed that all of the CD44^hi^CD62L^lo^CD4^+^ T cells from the mice were negative for CD103 (Fig 5C). Among them, a small, CD69^-^ portion (~20%) resembled the T_EM_ subset, but the majority of these (>70%) were CD69^+^, resembling a recirculating memory CD4^+^ T cell subset with the phenotype of CD44^hi^CD62L^lo^CD69^+^CD103^-^ (Fig 5C). The total cell number of CD103^-^CD69^+^ memory CD4^+^ T cells was significantly (P<0.01) increased by RB51 vaccination relative to the number seen in sPBS-dosed mice before and after wt challenge (Fig 5D). The CD103^-^CD69^+^CD4^+^memory T cells were even greater in znBAZ-vaccinated mice (P<0.05) relative to RB51-vaccinated mice before wt challenge. However, post-challenge levels of CD103^-^CD69^+^CD4^+^ memory T cells present in lungs from znBAZ-vaccinated mice were similar to those in the lungs from RB51-vaccinated mice (Fig 5D). No increased numbers of CD103^+^CD69^+^CD4^+^ memory T cells were detected for either vaccine group (S5 Fig). Further analysis was conducted to discern the phenotype of the recirculating memory CD4^+^ T cells. All the CD44^hi^CD62L^lo^CD69^+^ CD103^-^ memory CD4^+^ T cells expressed CD11a^hi^, but did not express CCR7, CD127, KLRG1, T-bet, granzyme B, nor perforin (Fig 5E). Examining their proinflammatory cytokine profiles revealed a 3-fold increase in IFN-γ^+^CD103^-^ CD4^+^ memory T cells by RB51- and 4-fold increase by znBAZ-vaccinated mice. Additionally, polyfunctional CD103^-^CD4^+^ memory T cells producing IFN-γ and TNF-α increased by 10-fold following RB51 or znBAZ vaccination (Fig 5F). All these results show that both znBAZ and RB51 are able to stimulate an influx of recirculating memory CD4^+^ T cells into the lungs bearing a memory phenotype expressing CD69 and CD11a, and are capable of producing proinflammatory cytokines.

### Mucosal znBAZ vaccination promotes enhanced recruitment of CD103^+^ and CD103^-^ CD8^+^ T_RM_ cells into the lungs

**Fig 6:**
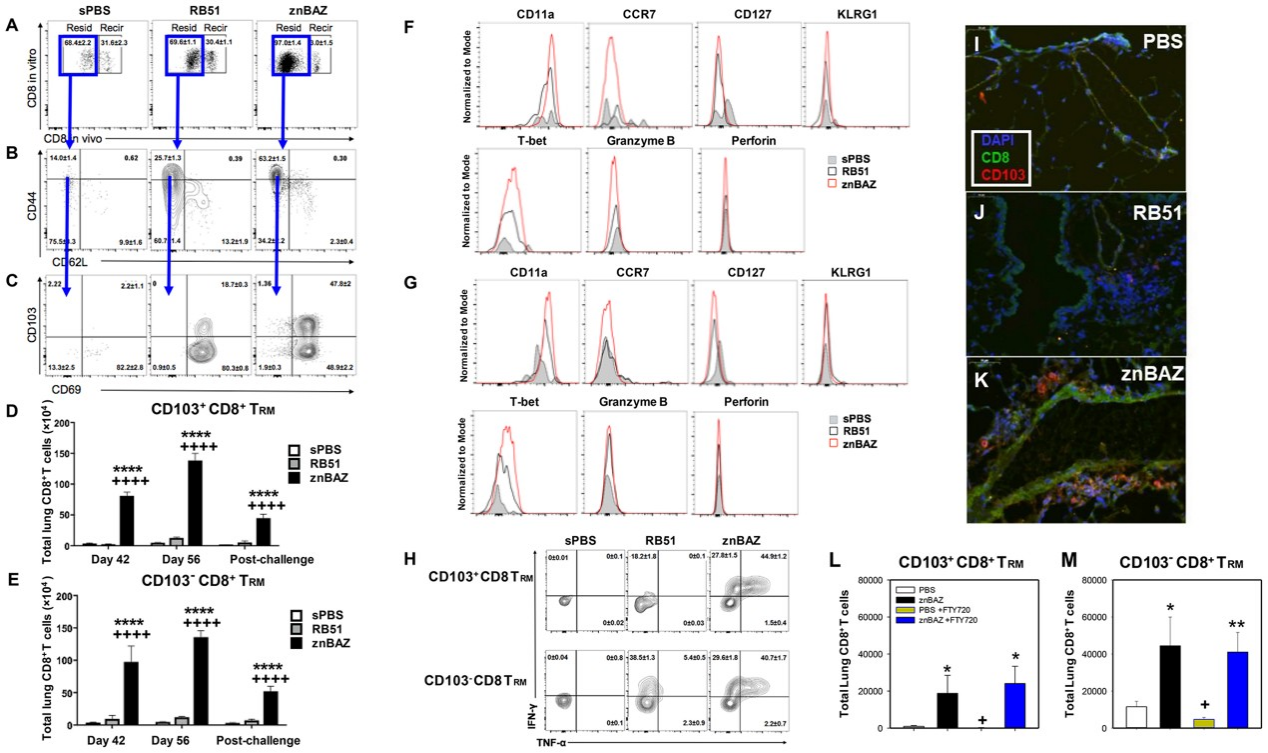
Mucosal znBAZ vaccination enhances the infiltration of CD103^+^ and CD103^-^ CD8^+^ T_RM_ cells into the lungs. BALB/c mice were primed and boosted with sPBS, RB51, and znBAZ as described in (Fig 1A). Mice were analyzed for the expression of memory CD8^+^ T cell subsets on days 42 and 56 (pre-challenge), as well as at day 84 (post-challenge with wt *B*. *abortus* 2308). (A) Flow cytometric analysis of lung CD8^+^ T cells following *in vivo* labeling on day 56. (B) FACS plots show the percentage of cells expressing CD62L and CD44, and (C) CD69 and CD103. (D, E) Total number of CD103^+^ and CD103^-^ CD69^+^ CD8^+^ T_RM_ cells in the lungs at various time points. The data depict n = 12 mice per group combined from three independent experiments. The differences were determined when compared to sPBS-dosed mice (****P<0.0001, ***P<0.001, **P<0.01, *P<0.05), or compared to RB51-vaccinated mice (^++++^P<0.0001, ^+++^P<0.001, ^++^P<0.01, ^+^P<0.05). The differences amongst the time points were determined when compared to day 42 (^††††^P<0.0001, ^†††^P<0.001, ^††^P<0.01, ^†^P<0.05), or compared to day 56 (^‡‡‡‡^ P<0.0001, ^‡‡‡^P<0.001, ^‡‡^P<0.01, ^‡^P<0.05). Analysis of variance with Two-way ANOVA Tukey’s multiple comparisons test was performed. (F-G) Representative expression of CD11a, CCR7, CD127, KLRG1, T-bet, granzyme B, and perforin by (F) CD103^+^ and (G) CD103^-^ CD8^+^ T_RM_ cells from the lung at day 56 after oral prime are depicted. (H) IFN-γ and TNF-α production by CD103^+^ and CD103^-^ CD8^+^ T_RM_ cells from the lungs on day 56 are shown. (I-K) Immunofluorescent staining for CD8^+^ T cells expressing CD103 reveals the presence of CD103^+^ and CD103^-^ CD8^+^ T_RM_ cells in the lungs of (K) znBAZ-vaccinated mice, and to a lesser extent in (I) PBS-dosed or (J) RB51-vaccinated mice. Magnification is 400x, and line depicts 50 μm in length. Data are representative of 3–5 mice per group. (L, M) Parenteral administration of FTY720 for one week beginning one day prior to nasal *B*. *abortus* 2308 challenge showed no significant change in (L) CD103^+^ and (M) CD103^-^ CD8^+^ T_RM_ cells relative to untreated znBAZ-vaccinated mice; **P < 0.01, *P < 0.05 versus respective PBS-dosed group and ^+^P < 0.05 between untreated PBS-dosed and FTY720-treated, PBS-dosed mice.

Pulmonary CD4^+^ T cells increase in mice vaccinated with either znBAZ or RB51 (Fig 2), but only znBAZ vaccination enhances CD8^+^ T cells in the lungs (Fig 2). Inquiring into the type of memory CD8^+^ T cells induced following znBAZ vaccination, fluorescently conjugated anti-CD8 mAb, given IV 10 min prior to CD8^+^ T cell isolation from the lungs, reveals the presence of recirculating and resident memory CD8^+^ T cells in mice immunized with znBAZ, RB51, or sPBS. Analysis of the lung lymphocytes was performed as has been done with CD4^+^ T_RM_ cells on days 42 and 56 (pre-challenge) as well as at day 84 (post-challenge). Fig 6A–6C depicts representative FACS plots at day 56 with CD8^+^ T cells showing mostly a resident memory phenotype. Two-thirds of the lung CD8^+^ T cells from sPBS- and RB51-vaccinated mice are resident cells (Fig 6A). Distinctively, znBAZ-vaccinated mice shows ~97% of the CD8^+^ T cells are resident lung, and only a minor portion are recirculating (Fig 6A). When gated on the resident CD8^+^ T cells, naïve mice show only a minor portion (14%) exhibiting a memory phenotype as CD44^hi^CD62L^lo^ (Fig 6B). Conversely, in RB51- and znBAZ-vaccinated mice, the proportions of CD44^hi^CD62L^lo^ memory CD8^+^ T cells are increased by 2-fold and 4.5-fold, respectively (Fig 6B). All of the CD8^+^ T_RM_ cells from vaccinated mice express CD69, and interestingly, half of the CD69^+^ CD8 T_RM_ cells expresses CD103^+^ following znBAZ immunization, which is 2.5-fold more than seen in RB51-vaccinated mice (Fig 6C). The number of lung CD103^+^CD8^+^ T_RM_ cells is increased by 32-fold at day 42, 36-fold at day 56, and 20-fold post-challenge in znBAZ-vaccinated mice compared to sPBS-dosed mice (Fig 6D). Very similar numbers are observed for the influx of CD103^-^CD8^+^ T_RM_ cells (Fig 6E). The enhanced number of CD103^+^ and CD103^-^ CD8 T_RM_ is unique for znBAZ-vaccinated, i.e., not seen in RB51-vaccinated mice. Previous reports showed that T_RM_ cells express CD11a, but not CCR7 [27,30]. This work shows similar findings as evidenced by both CD103^+^ and CD103^-^ CD8 T_RM_ cells being CD11a^high^, not CCR7^+^ (Fig 6F and 6G). Moreover, these do not express CD127, KLRG1, granzyme B, or perforin, but T-bet expression was increased (Fig 6F and 6G). Immunofluorescent staining of whole lung sections confirmed the presence of T_RM_ cells in znBAZ-vaccinated mice, while minimal to no T_RM_ cells in RB51-vaccinated or PBS-dosed mice (Fig 6I–6K). Both CD103^+^ and CD103^-^ CD8^+^ T_RM_ cells are detected in lungs from znBAZ-vaccinated mice (Fig 6K), and all of these were CD44^+^ (S6C Fig), while minimal to none of these CD44^+^ CD8^+^ T_RM_ cells being observed in lungs from PBS-dosed or RB51-vaccinated mice (S6A and S6B Fig). To further confirm that the lung CD8^+^ T cells from znBAZ-vaccinated mice are T_RM_ cells, in vivo treatment with FTY720 was performed to determine if these CD8^+^ T cell levels are reduced in the lungs. Groups of znBAZ-vaccinated and PBS-dosed mice were given daily administrations of FTY720 for one week beginning one day prior to nasal challenge with wild-type *B*. *abortus* 2308. The number of lung CD103^+^ and CD103^-^ CD8^+^ T_RM_ cells in znBAZ-vaccinated mice was not affected by FTY720 treatment relative to untreated, znBAZ-vaccinated mice (Fig 6L and 6M), only FTY720-treated, PBS-dosed mice showed reduced levels of lung CD8^+^ T cells (P<0.05).

With respect to cytokine production, high proportions of IFN-γ^+^ and polyfunctional IFN-γ^+^TNF-α^+^ CD103^+^ and CD103^-^ CD8^+^ T_RM_ cells following znBAZ vaccination were observed. Greater than 27% of CD103^+^ CD8^+^ T_RM_ cells and CD103^-^ CD8^+^ T_RM_ cells were IFN-γ^+^, and >40% of CD103^+^ CD8^+^ T_RM_ cells and CD103^-^ CD8^+^ T_RM_ cells produced both IFN-γ and TNF-α (Fig 6H). Collectively, these results demonstrate that mucosal znBAZ vaccination enhances the influx of lung IFN-γ^+^ and polyfunctional CD103^+^ and CD103^-^ CD8^+^ T_RM_ cells implicating their protective role against pulmonary wt *B*. *abortus* infections.

### Mucosal znBAZ vaccination elicits distinct CXCR3^+^ CD8^+^ T_RM_ cell subsets that reside in lung parenchyma and airways

**Fig 7:**
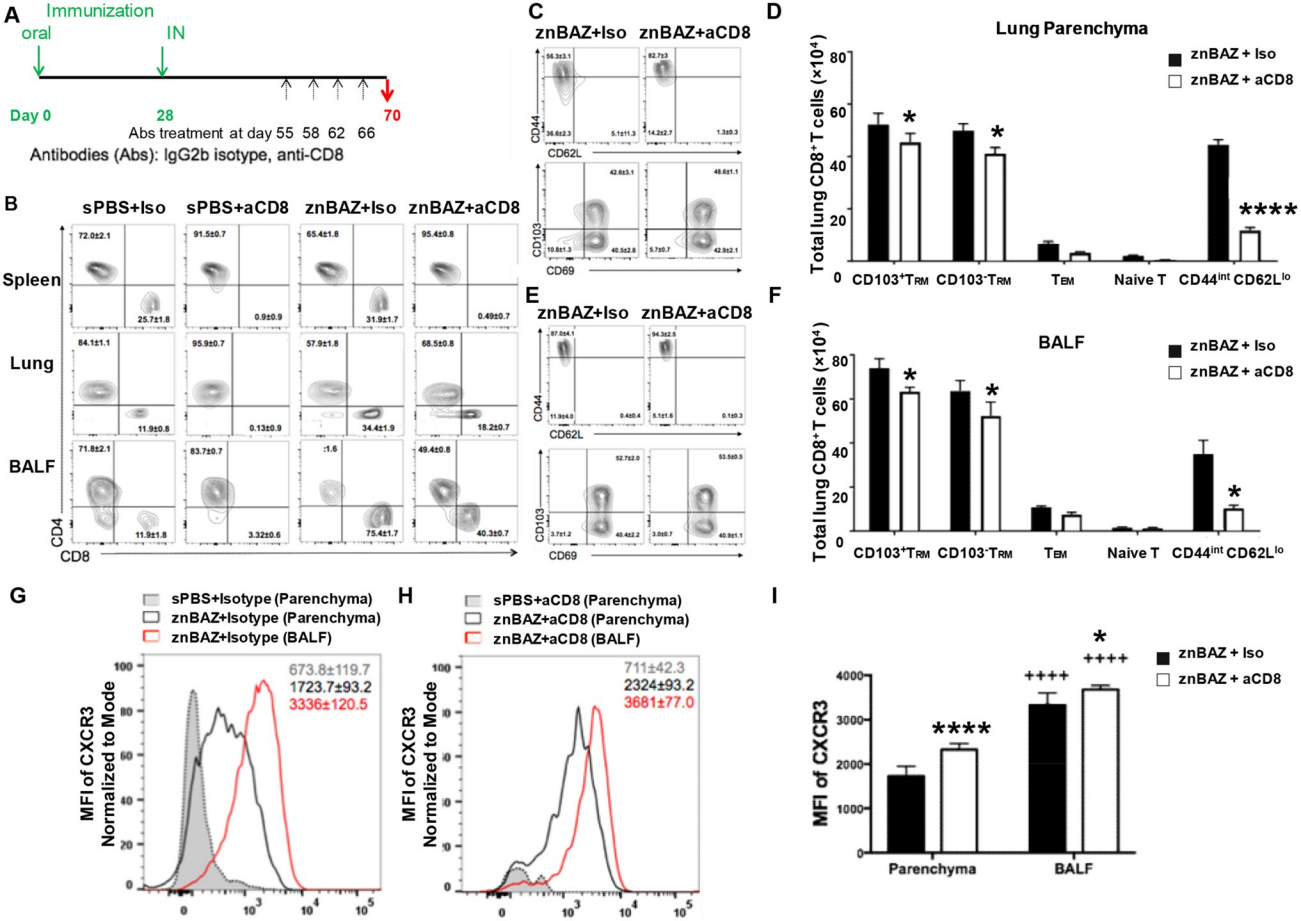
Mucosal znBAZ vaccination elicits CXCR3^lo^ and CXCR3^hi^, CD8^+^ T_RM_ cell subsets that are enriched in lung parenchyma and BALF. (A) BALB/c mice were primed/boosted with sPBS or znBAZ as depicted. On days 55, 57, 62, and 66, mice were treated IP with isotype or anti-CD8 mAb. On day 70, mice were evaluated for extent CD8^+^ T cell infiltration into the lung parenchyma and airways without wt challenge. (B) Flow cytometry analysis of CD4^+^ and CD8^+^ expression on TCR-β^+^ cells from spleen, lung parenchyma, and BALF following isotype or anti-CD8 mAb treatment in sPBS- and znBAZ-vaccinated mice. FACS plots show the expression of CD44, CD62L, CD103, and CD69 on CD8^+^ T cells from (C) lung parenchyma and (E) BALF. Total CD103^+^CD8^+^ T_RM_, CD103^-^CD8^+^ T_RM_, T_EM_, naïve T cells, and CD44^int^CD62L^lo^ CD8^+^ T cells are shown from the (D) lung parenchyma and (F) BALF. The data depict n = 12 mice per group combined from three independent experiments; the difference between isotype and anti-CD8 mAb was determined by ****P<0.0001, ***P<0.001, **P<0.01, *P<0.05. The differences between lung parenchyma and BALF were determined to be significant: ^++++^P<0.0001, ^+++^P<0.001, ^++^P<0.01, ^+^P<0.05; analysis of variance with Two-way ANOVA Tukey‘s multiple comparisons test. (G-H) Histograms of CXCR3 expression by CD8^+^ T cells from the lung parenchyma and BALF are depicted of mice treated with (G) isotype or (H) anti-CD8 mAb, and (I) their respective MFIs are depicted; the differences between isotype and anti-CD8 mAb are indicated as ****P<0.0001, *P<0.05; and the differences between lung parenchyma and BALF are indicated as ^++++^P<0.0001.

The lung parenchyma and airways comprise an important immunological niche to defend against pulmonary infections [43,47,61,62]; hence, both lung parenchyma and bronchoalveolar fluid (BALF) were evaluated. To ascertain which portion of CD8^+^ T cells are resistant to anti-CD8 mAb depletion, groups of BALB/c mice were primed and boosted with znBAZ as described above, or dosed with sPBS. Beginning on day 56, mice were subjected to four anti-CD8α mAb or IgG2b isotype Ab treatments over a period of two weeks (Fig 7A). The extent of CD8^+^ T cell depletion was confirmed by FACS analysis of peripheral blood lymphocytes. The anti-CD8 mAb depletion regimen was effective in eliminating the splenic CD8^+^ T cells (Fig 7B top row). A little less than half of the pulmonary CD8^+^ T cells from the znBAZ-vaccinated mice were resistant to depletion in the lung parenchyma (Fig 7B middle row) and in the BALF (Fig 7B bottom row). Notably, CD8^+^ T cells dominated in the BALF representing 75.4% of TCR-β^+^ cells, and 34.4% of CD8^+^ T cells were found in lung parenchyma (Fig 7B). These results support data from the post-challenge study (Fig 4) showing that anti-CD8 mAb treatment is unable to deplete the pulmonary CD8^+^ T cells in znBAZ-vaccinated mice, and these CD8^+^ T cells are retained in lung parenchyma and airways.

To interrogate which lung CD8^+^ T cells are eliminated by anti-CD8 mAb treatment, Ag-specific memory CD8 T cells with an intermediate CD44 (CD44^int^) expression have been previously described [63–65]. Lung parenchyma CD44^int^CD62L^lo^CD8^+^ T cells from znBAZ-vaccinated mice subjected to mAb treatment are sensitive to depletion exhibiting reduced levels (14.2%) compared to isotype control-treated group (36.6%). Consequently, the anti-CD8 mAb treatment nets an increase in the percentage of CD44^hi^CD62L^lo^ memory CD8^+^ T cells from 56% to >82% (Fig 7C top row). Within the CD44^hi^CD62L^lo^ fraction, the CD69^-^ population is reduced nearly in half by anti-CD8 mAb treatment (Fig 7C bottom row). However, the ratio of CD103^+^/CD103^-^ lung memory CD8^+^ T cells in znBAZ-vaccinated mice does not change between groups (Fig 7C bottom row). Anti-CD8 mAb treatment only slightly reduces (by 15%) the total number of CD103^+^ or CD103^-^ CD8^+^ T_RM_ cells present in the lung parenchyma, and does not change the ratio of CD103^+^ to CD103^-^ CD8^+^ T cells (Fig 7D). In contrast, the total number of CD44^int^ CD62L^lo^ CD8^+^ T cells is significantly reduced (P<0.0001) by ~4-fold (Fig 7D). Anti-CD8 mAb treatment minimally impacts the proportion (Fig 7C) or total number of CD8^+^ T_EM_ (CD44^hi^CD62L^lo^ CD69^-^) and naïve CD8^+^ T cell (CD44^lo^CD62L^hi^) subsets (Fig 7D). Applying similar analyses to BALF lymphocytes from znBAZ-vaccinated mice (Fig 7E and 7F), anti-CD8 mAb treatment significantly (P<0.05) reduces the proportion of CD44^int^CD62L^lo^ CD8^+^ T cells by more than half to 5.13%. The anti-CD8 mAb treatment does not alter CD8^+^ T_EM_ and naïve CD8^+^ T cell subset percentages (Fig 7E) nor their cell numbers (Fig 7F). The ratio of BALF CD103^+^/CD103^-^ CD8^+^ T cells also does not change by anti-CD8 mAb treatment (Fig 7E bottom row), but their total number is slightly reduced by ~16% (P<0.05). Again, the majority of CD103^+^ or CD103^-^ CD8^+^ T_RM_ cells are retained in the lung airways (Fig 7F). These results demonstrate that the CD44^int^CD62L^lo^ CD8^+^ T cells are more sensitive to anti-CD8 mAb treatment than the CD103^+^ or CD103^-^ CD8^+^ T_RM_ cells.

Subsequent analysis examines the lung parenchyma and the airway CD8^+^ T cells for expression of CXC-chemokine receptor 3 (CXCR3), and whether *in vivo* anti-CD8 mAb treatment affects CD8^+^ T cell localization in the lungs. CXCR3 is required for differentiation and localization of T_RM_ cells to mucosal tissues such as to the epithelium of lung, small intestine, and skin [27,32,34,47,66]. The airway CD8^+^ T cells recovered from the BALF of znBAZ-vaccinated mice treated with isotype Ab expressed CXCR3^hi^ with a mean fluorescence intensity (MFI) of 3336 ± 120.5, which is significantly greater (P<0.0001) than those CXCR3^lo^ CD8^+^ T cells retained in the lung parenchyma with a MFI of 1723.7 ± 93.2 (Fig 7G and 7I). The MFI for CXCR3 from the sPBS-dosed mice treated with isotype Ab control is only 673.8 ± 119.7 (Fig 7G). These data show that cells from lung parenchyma and airway express distinct levels of CXCR3 following znBAZ vaccination. The anti-CD8 mAb treatment does not affect CXCR3 expression in lung parenchyma and lung airways relative to isotype treatment (Fig 7G and 7I). Very few CD8^+^ T cells are observed in the sPBS-dosed mice following anti-CD8 depletion (Fig 7H). Additionally, the majority of CD103^+^ (> 80%) and CD103^-^ (> 76%) CD8^+^ T_RM_ cells express CXCR3^lo^ in the lung parenchyma (S6A Fig) and more than 95% of the airway CD103^+^ and CD103^-^ CD8^+^ T_RM_ cells express CXCR3^hi^ (S6B Fig). The expression patterns for CXCR3^lo^ and CXCR3^hi^ by CD103^+^ and CD103^-^ CD8^+^ T_RM_ cells do not change by the anti-CD8 mAb treatment (S6 Fig). Furthermore, almost all of the CD8^+^ T cells induced by znBAZ immunization belong to the resident phenotype and remain in the lung parenchyma and airways despite anti-CD8 mAb treatment (S7 Fig). These data show that mucosal znBAZ vaccination increases the presence of CD8^+^ T_RM_ cells expressing CXCR3^lo^ and CXCR3^hi^ phenotypes in the lung parenchyma and airways, and *in vivo* anti-CD8 mAb treatment increases the MFI for CXCR3 on those CD8^+^ T_RM_ cells resistant to depletion.

### In vivo IL-17 neutralization treatment has no impact upon znBAZ efficacy

**Fig 8:**
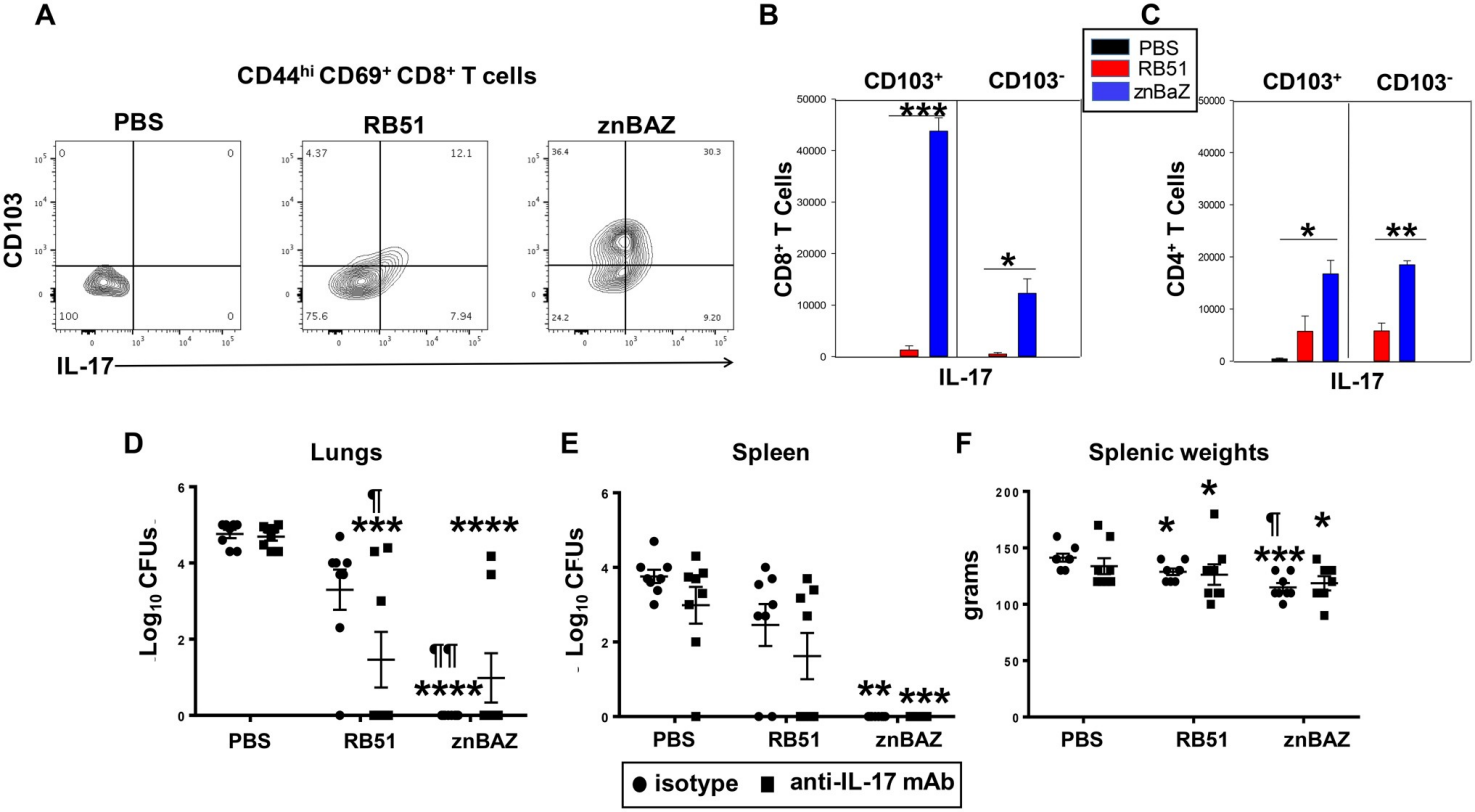
znBAZ induces IL-17 production, but not required for protection against wt *B*. *abortus* challenge. Groups of BALB/c mice were orally primed, nasally boosted with sPBS, RB51, or znBAZ as described in Fig 1A, and on day 42 post-primary immunization, gated lung CD44^hi^ CD69^+^ CD8^+^ T_RM_ cells were evaluated (A, B) to determine the source of IL-17 production by CD103^+^ and CD103^-^ T_RM_ subsets. The total lung CD103^+^ and CD103^-^ (B) CD8^+^ T_RM_ cells and (C) CD4^+^ T_EM_ cells producing IL-17 were quantified; ***P<0.001, **P<0.01, *P<0.05, significant differences against sPBS-dosed or RB51-vaccinated mice are shown analysis of variance with One-way ANOVA Tukey‘s multiple comparisons test. (D-F) Additional groups of BALB/c mice were vaccinated in the same manner, and then challenged with virulent *B*. *abortus* 2308 as described above. Half of all mice were treated with isotype control Ab and the other half with anti-17 mAb on days -1, 2, 7, and 12 post-challenge. Two weeks after challenge, extent of brucellae colonization of the (D) lungs and (E) spleens were measured. (F) Splenic weights were also measured. Analysis of variance with Two-way ANOVA Tukey’s multiple comparisons test was performed: ****P<0.0001, ***P<0.001, **P<0.01, *P<0.05, versus isotype control-treated sPBS-dosed mice; ^¶¶^P<0.01, ^¶^P<0.05 versus isotype control-treated RB51-vaccinated mice. Data depict results from two experiments.

Studies sought to understand the role of IL-17 induced by znBAZ vaccination. Groups of BALB/c mice were orally primed, nasally boosted with sPBS, RB51, or znBAZ as described above. On day 42 post-primary immunization, the relative distribution of lung IL-17-producing CD103^+^ and CD103^-^ CD8^+^ T_RM_ cells was measured by flow cytometry (Fig 8A–8C). IL-17 was notably elevated for znBAZ-vaccinated mice relative to RB51-vaccinated or sPBS-dosed mice. Inquiring further into the source of this IL-17 derived from znBAZ-vaccinated mice, ~2.6-fold more IL-17 came from lung CD8^+^ T_RM_ cells relative to CD4^+^ T_EM_ cells (Fig 8B and 8C). The IL-17 was 3.6-fold more from the CD103^+^ CD8^+^ T_RM_ cells versus CD103^-^ CD8^+^ T_RM_ cells. The number of IL-17-producing CD8^+^ CD103^+^ T_RM_ cells greatly exceeded those induced by RB51 vaccination (P < 0.001) by 34-fold. For CD4^+^ T_RM_ cells, IL-17 production was equally distributed between the CD103^+^ and CD103^-^ subsets, and both significantly exceeded (P < 0.05) the respective CD4^+^ T_RM_ subsets present in RB51-vaccinated mice (Fig 8C).

Given the elevated presence of IL-17-producing T_RM_ cells in the lungs from znBAZ-vaccinated mice, we queried the relevance of these cells for protection against virulent *B*. *abortus* 2308 challenge. Additional groups of BALB/c mice were orally primed, nasally boosted with sPBS, RB51, and znBAZ as described above. IL-17 was neutralized in vivo by administering an anti-mouse IL-17 mAb or its isotype control over a two-wk course of treatments beginning on day 55 during wt *B*. *abortus* challenge. The extent of wt *B*. *abortus* colonization of the lungs and spleens was evaluated as a measure of vaccine efficacy (Fig 8D–8F). No significant reduction of brucellae colonization of the lungs or the spleens of znBAZ-vaccinated mice as a consequence of in vivo IL-17 neutralization was seen compared to isotype control-treated mice. Hence, in vivo IL-17 neutralization had no significant impact on protection conferred by znBAZ vaccination relative to sPBS-dosed groups. In contrast, IL-17 neutralization did significantly enhance RB51’s efficacy (P < 0.05) as noted by the reduced brucellae present in the lungs (Fig 8D). Anti-IL-17 mAb treatment had no impact upon brucellae colonization of the spleen in RB51-vaccinated mice (Fig 8E). Isotype control-treated RB51-vaccinated mice showed no significant difference in lung colonization relative to isotype control-treated sPBS-dosed mice (Fig 8D).

### Superior protective potency conferred by lung CD8^+^ T_RM_ cells following mucosal znBAZ vaccination

**Fig 9:**
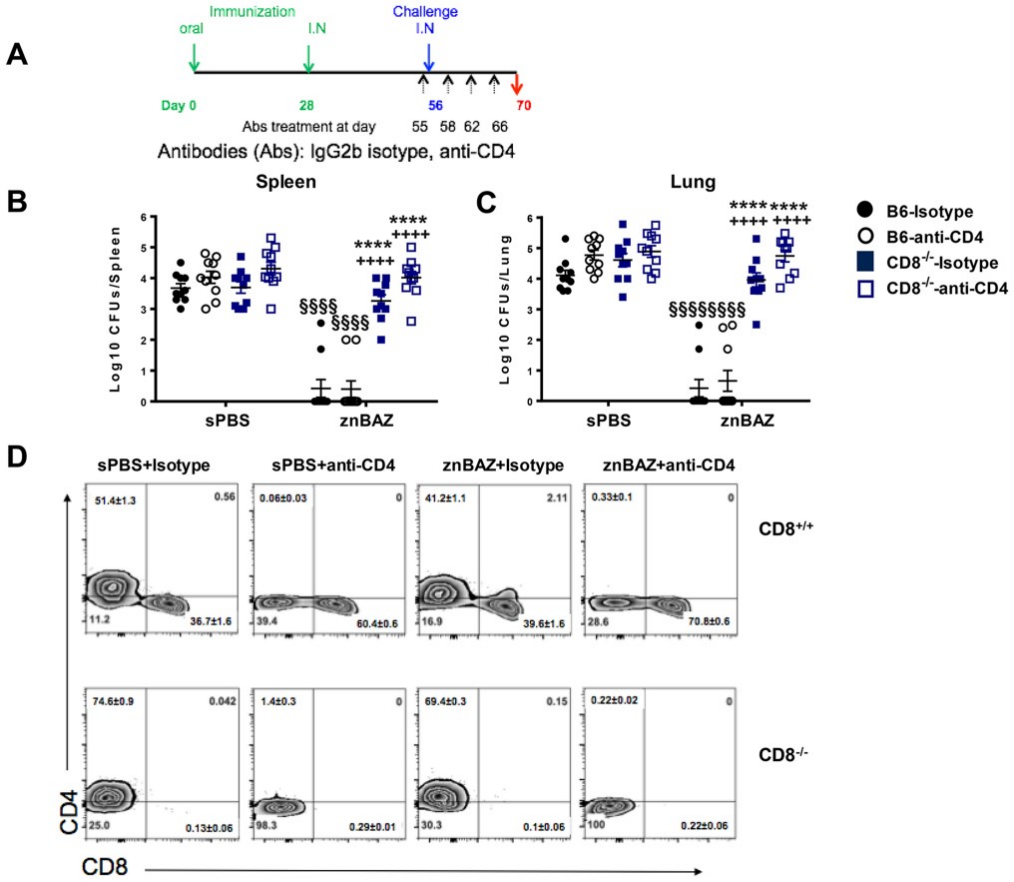
Superior protective efficacy conferred by lung CD8^+^ T_RM_ cells following mucosal znBAZ vaccination. (A) C57BL/6 (B6) and C57BL/6 CD8^-/-^ (CD8^-/-^) mice were orally primed and nasally boosted with sPBS and znBAZ as depicted. On day 56, all mice were challenged with *B*. *abortus* 2308, and on days 55, 57, 62, and day 66, mice were treated IP with isotype control or anti-CD4 mAb. On day 70, all mice were assessed for extent of colonization by wt *B*. *abortus* 2308 in (B) lungs and (C) spleens. (D) Flow cytometry analysis of CD4^+^ and CD8^+^ expression from TCR-β^+^ cells in the lungs post-challenge following isotype or anti-CD4 mAb treatment for each vaccination group in B6 and CD8^-/-^ mice was assessed (n = 10 mice per group). The data depict the mean ± SEM of two independent experiments; the differences were determined when compared to isotype control-dosed B6 mice (****P<0.0001), or compared to anti-CD4 mAb-treated B6 mice (^++++^P<0.0001). The difference between sPBS and znBAZ immunization groups are indicated (^§§§§^P<0.0001). Analysis of variance with Two-way ANOVA Tukey’s multiple comparisons test was done.

Since pulmonary CD8 T_RM_ cells are resistant to mAb depletion, an alternate approach is needed to determine whether CD8^+^ T_RM_ cells are required for protection against pulmonary wt *B*. *abortus* 2308 challenge. To this end, B6 and B6 CD8^-/-^ (CD8^-/-^) mice were orally primed with sPBS or znBAZ on day 0, and nasally boosted on day 28. Four weeks after boost, all mice were challenged with virulent *B*. *abortus* 2308, and on day 55 (one day before challenge), half of the C57BL/6 CD8^-/-^ mice were treated with anti-CD4 mAb or isotype control. Additional mAb treatments were given on days 57, 62, and 66 post-challenge (Fig 9A). Evaluation of peripheral blood CD4^+^ T cell levels by FACS analysis confirmed that the depletion was effective. Two weeks after challenge (day 70), lungs and spleens were evaluated for the extent of wt *B*. *abortus* 2308 colonization and from their T cell profiles. Colonization of the lungs (Fig 9B) and spleens (Fig 9C) from naïve (sPBS-dosed) mice was not significantly affected by either anti-CD4 mAb treatment or by being deficient of CD8^+^ T cells (CD8^-/-^ mice). Mucosally znBAZ-vaccinated B6 mice treated with or without anti-CD4 mAb maintained protection against wt challenge evidenced by the > 4 log reduction in brucellae in both lungs and spleens relative to sPBS-dosed mice (Fig 9B and 9C). However, znBAZ’s efficacy was abated in CD8^-/-^ mice (Fig 9B and 9C). CD4^+^ T cell depletion resulted in a slight CFU increase, but not significantly in either the CD8^-/-^ lungs nor spleens (Fig 9B and 9C). This absence of efficacy was confirmed by lung T cell analyses. The results showed that anti-CD4 mAb neutralized all of the lung CD4^+^ T cells in sPBS-dosed and znBAZ-vaccinated mice (Fig 9D). These results demonstrate that CD8^-/-^ mice lost protection from mucosal znBAZ vaccination because of the absence of CD8^+^ T_RM_ cells. Together with the results from Fig 4, we conclude that the CD8^+^ T_RM_ cells in znBAZ-vaccinated mice are critical for protection against virulent pulmonary challenge with wt *B*. *abortus* 2308.

## Discussion

*Brucella* infections primarily occur following a mucosal exposure to contaminated aerosols or ingestion of contaminated foods. During infection, the pathogens are transported across the mucosal epithelium and endocytosed by mucosal macrophages and DCs, and then disseminate, resulting in a systemic disease of the reticuloendothelial system [3]. With this in mind, a logical approach for protection against *Brucella* is to develop an effective vaccine that protects both the mucosal and systemic immune compartments [67,68]. Following mucosal immunization, Ag-triggered B and T cells leave the draining LNs, transit through the lymph, enter the blood circulation and then enter the mucosal tissues [67–70]. Protective mucosal immune responses are most effectively induced by mucosal immunization through oral, nasal, or rectal routes [67]. Thus, our study adopted a prime-boost vaccination strategy using a combination of oral and nasal administrations. Unlike the conventional RB51 vaccine, nearly sterile protection was conferred against pulmonary wt *B*. *abortus* challenge in znBAZ-vaccinated mice as evidenced by the clearance of brucellae from their lungs and spleens.

For these studies, we elected to use the rough RB51 vaccine, which is most commonly used for vaccination against brucellosis. We have done comparison studies in mice, and do not find differences in protection between the smooth *B*. *abortus* vaccine strain 19 (S19) and RB51. Nor do we observe a change in T cell bias, namely that CD4^+^ T cells are predominantly induced following an oral-prime, nasal-boost vaccination regimen (manuscript in preparation). S19’s efficacy was similar to that of RB51. Hence, protection conferred by znBAZ is not solely attributed to being a smooth vaccine, but is associated more with its altered pathogenicity as a result of the absence of *znuA* and *norD* genes. Despite its difference from RB51, the znBAZ clearance rate is similar to that of RB51, while S19 is retained longer and not cleared as effectively from the host [19]. The protection conferred by S19 is equivalent to RB51, and also found to be CD4^+^, not CD8^+^ T cell-dependent, and S19 was not able to confer complete protection as does znBAZ (manuscript in preparation).

What is seminal about the presented data regarding znBAZ vaccination is the demonstration that protection is solely CD8^+^ T cell-dependent. Past studies examining host immunity to *Brucella* infections have shown the essential role for IFN-γ-producing CD4^+^ T cells when exposed wt *Brucella* [11,12,14,21], vaccine components [13,14], or attenuated strains [23,71]. CD8^+^ T cells were assumed to be less important for protection against brucellosis than CD4^+^ T cells, and CD8^+^ T cell immunity was thought to be limited to cytotoxicity [11,15,20,72,73]. In fact, CD8^-/-^ mice orally vaccinated with the attenuated *B*. *melitensis* WR201 strain showed no loss of protection against pulmonary challenge with virulent wt *B*. *melitensis* demonstrating that CD8^+^ T cells are not crucial for protection from pulmonary *Brucella* infection [74]. To interrogate the relevance of the induced T cell subsets, various studies were undertaken to define the mechanism of protection. These involved flow cytometric analysis of T cells induced following mucosal vaccination, and their induced cytokine responses. In vivo labeling studies clearly pointed to the development of CD8^+^ T_RM_ cells, and these were found to be refractive to *in vivo* CD8 T cell depletion. Only the complete absence of CD8^+^ T cells in CD8^-\-^ mice prevented the znBAZ vaccine from being effective. Mice mucosally vaccinated with znBAZ and depleted of their CD4^+^ T cells remained fully protected against virulent challenge, diminishing the relevance of CD4^+^ T cells for znBAZ-induced protection. As demonstrated here, protection conferred by RB51 was CD4^+^ T cell-dependent since their depletion abated their protective capacity. In contrast, the presented results demonstrate that znBAZ vaccination elicits CD8^+^ T cells to confer full protection. Examination of their effector function revealed the elevated production of IFN-γ and TNF-α and presence of IFN-γ^+^, TNF-α^+^, and polyfunctional CD8^+^ T_RM_ cells. Following challenge with virulent *B*. *abortus* 2308, the total numbers of CD8^+^ T_RM_ cells were reduced to ~one-third the levels present pre-challenge, and the total numbers of IFN-γ^+^, TNF-α^+^, and polyfunctional CD8^+^ T cells were reduced to one-third to one-half of pre-challenge levels. Interestingly, IL-17 was highly induced by znBAZ, and derived mostly from CD8^+^ T_RM_ cells, although a significant portion was produced by the memory CD4^+^ T cells; and IL-17-producing CD8^+^ T_RM_ cells were not reduced post-challenge. Despite eliciting elevated levels of IL-17-producing CD8^+^ T_RM_ cells in the lungs, in vivo IL-17 neutralization had no impact on brucellae colonization of the lungs or the spleen in znBAZ-vaccinated mice. In contrast, RB51 vaccination resulted in dramatically less IL-17, and upon its neutralization, brucellae colonization of the lungs was reduced. In fact, the number of IL-17-producing CD8^+^ T_RM_ cells exceeded those induced in the lungs following RB51 vaccination by 34-fold. Yet, IL-17 neutralization of RB51-vaccinated mice did have a significant impact resulting in ~430-fold reduction in lung colonization following challenge, making it apparent that IL-17’s impact is greater for mice vaccinated with RB51 rather than those vaccinated with znBAZ. Such finding points to possible alternative mechanisms for IL-17 in znBAZ-vaccinated mice. IL-17 has been shown to suppress IFN-γ [75–77], which is highly induced in znBAZ-vaccinated mice. Such elevation suggests that the induced IL-17 may regulate this Th1-type response. Future studies will probe into how this IL-17 is induced, and determine its regulatory potential.

Examination for other effector molecules revealed no change in perforin or granzyme B levels when compared to those in RB51-vaccinated mice. Likewise, no differences were observed for the memory CD4^+^ T cells. These results point to CD8^+^ T_RM_ cells being the primary source of cells responsible for the elimination of wt *B*. *abortus*. We speculate that the failure of RB51 to induce either CD4^+^ or CD8^+^ T_RM_ cells results in the diminished capacity to protect against pulmonary challenge with virulent *B*. *abortus*. We also speculate that ability to induce CD8^+^ T_RM_ cells is linked to znBAZ’s defined genetic deficiencies since mice mucosally dosed twice with RB51 lacked the capacity to elicit CD4^+^ or CD8^+^ T_RM_ cells.

*In vivo* T cell depletion studies found that the limited protection conferred by RB51 immunization was completely abrogated in spleens and lungs upon CD4^+^ T cell depletion, but not in CD4^+^ T cell-depleted znBAZ-vaccinated mice, which remained refractive to nasal challenge. These findings further implicate the importance of CD4^+^ T cells for protection conferred by RB51, but not by znBAZ. RB51-vaccinated mice depleted of their CD8^+^ T cells showed no significant reduction in protection, further suggesting that CD8^+^ T cells contribute less to protection than do CD4^+^ T cells. Interestingly, when a similar technique was applied to eliminate CD8^+^ T cells from znBAZ-vaccinated mice, efficacy was only modestly affected in the lungs, further implicating the importance of lung CD8^+^ T cells for znBAZ-mediated protection. Being refractive to anti-CD8 mAb treatment pointed to a mechanism whereby tissue-bound or resident memory T cells are induced. The *in vivo* anti-CD8 mAb treatment was not defective since CD8^+^ T cells were depleted in the spleens and lungs of sPBS-dosed and RB51-vaccinated mice, as well as being depleted in the spleens of znBAZ-vaccinated mice. Subsequent inquiries by *in vivo* labeling studies into the nature of the lung CD8^+^ T cells revealed that these cells are lung resident T cells since these were refractive to labeling by the anti-CD8 mAb.

Tissue-resident memory T cells maintain residence in peripheral tissues including the respiratory tract, the intestinal epithelium, epidermis, salivary glands, and central nervous system without recirculating through blood or lymph, even after an infection is cleared [27,30]. The lack of recirculation via the bloodstream is the key parameter in defining the term ‘tissue resident’. Tissue-resident T cells are the ones that undergo little or no recirculation, as opposed to T cells that are only transiently present within a tissue [27,43,78]. Thus, IV *in vivo* labeling methods are unable to access or neutralize T_RM_ cells [31,44]. Such an *in vivo* labeling method has been previously used to distinguish between leukocyte subsets in recirculation from resident T cells in the lungs [45–48]. Using this method, lung parenchymal and airway cells will stain *ex vivo* with the mAbs specific to CD4^+^ and CD8^+^ T cells, labeling both circulating and resident T cells, with resident cells within the lung parenchyma and airways are refractive to *in vivo* labeling [45,46]. With this method, both a transient, circulating population of memory CD4^+^ T cells and distinct resident memory CD8^+^ T cells were identified in mucosally znBAZ-vaccinated mice. In contrast, 99% of the CD4^+^ T cells from the mice vaccinated with RB51, znBAZ, or sPBS were confirmed as circulating CD4^+^ T cells, being sensitive to *in vivo* neutralization (Fig 4). Analysis of memory T cell subsets were found to be memory CD4^+^ T cells similar to T_RM_ cells rather T_CM_ or T_EM_ cells since the expression of CD69^+^ and CD11a^hi^ was upregulated, and negative for CCR7. CD69 is as a marker of T_RM_ cells, and is expressed on T_RM_ cells in skin, lungs, and the GI tract [33,34,36,46,79,80]. CD69 is a marker of recently activated T cells in LNs [81], which can be temporarily upregulated on T cells of any subset following restimulation [82]. Factors that influence CD69 upregulation include TNF-α and IFN-γ [46,83,84]. The fact that both znBAZ and RB51 are able to enhance the production of TNF-α and IFN-γ may be the reason for increased expression of CD69 by circulating memory CD4^+^ T cells. The integrin CD103, another marker of T_RM_ cells, has more dominant expression on CD8^+^ T_RM_ cells than on CD4^+^ T cells [34,85]. CD11a, the α-chain of the α_L_β2 integrin, plays a critical role in the homing of lymphocytes across high endothelial venules to enter LNs [86]. T_RM_ cells are known to upregulate the expression of CD11a [30]. CCR7 is involved in homing of various subpopulations of T cells and dendritic cells (DCs) to the LNs [87]. Reports indicate that CCR7 is required for CD4^+^ T cells migration from mouse skin to afferent lymphatics, and that blocking CCR7 expression prevents T cells from leaving the skin [88,89]. Expression of CCR7 was observed on a T cell population that migrated out of human skin, while CCR7^−^ T cells persisted in skin as did T_RM_ cells [90]. Thus, we used these basic cellular markers to define the T_RM_ cells at play.

Although non-recirculating populations of CD4^+^ T_RM_ cells have been described, CD8^+^ T_RM_ is the best-defined population [31–38]. Respiratory CD8^+^ T_RM_ cells serve as a first line of defense against pathogen challenge in the lungs and limit early viral replication [50–53]. Their beneficial role following bacterial infections is beginning to be appreciated [91]. Recent work has highlighted their ability to rapidly produce proinflammatory cytokines, IFN-γ and TNF-α, but not granzyme B or perforin, being critical for CD8^+^ T_RM_ cells protecting against reencountered pathogens [35,56,91]. The present study shows that mucosal vaccination with znBAZ, but not RB51, enhances the recruitment of polyfunctional CD8^+^ T cells to lungs (Fig 2) with 97% of them being tissue-resident CD8^+^ T cells. Additionally, the majority of these resident CD8^+^ T cells are memory (CD44^hi^CD62L^lo^) T cells, exhibiting increased expression of T_RM_ cell markers including CD69 and CD11a. Half of them express CD103 and others are CD103^-^ T_RM_ cells [27,90]. One study suggest these different T_RM_ cells are functionally unique in that CD103^−^CD8^+^ T_RM_ cells generated as a consequence of gut inflammation are different from CD103^+^ CD8^+^ T_RM_ cells, in their ability to control infection [33]. Immunofluorescence data further support the notion that indeed these CD103^+^ and CD103^-^ CD8^+^ T cells are T_RM_ cells as these were found residing in the bronchio-alveolar regions. Subsequent analysis revealed these being CD44^=^. In vivo treatment with FTY720 also demonstrated no reduction in lung CD103^+^ and CD103^-^ CD8^+^ T_RM_ cells in vaccinated mice confirming that pulmonary T_RM_ cells mediate protection against pulmonary challenge with wild-type *B*. *abortus*.

The CD8^+^ T_RM_ cells expressing CD103^+^ or CD103^-^ exhibited similar polyfunctional capacity to produce IFN-γ and TNF-α, but lack the cytotoxic activity of granzyme B and perforin. The expression patterns of CD127 (IL-7Rα) and KLRG1 (killer-cell lectin-like receptor G1) have been used to define the phenotype of the memory precursor CD8^+^ T cells [92,93]. CD127^hi^ KLRG1^low^ are memory precursor effector cells, whereas CD127^lo^ KLRG1^hi^ are short-lived, effector CD8^+^ T cells. However, less is known about the expression pattern of CD127 and KLRG1 on T_RM_ cells. In this present study, CD127 and KLRG1 expression by T_RM_ cells was examined. Precursors of T_RM_ cells express low levels of KLRG1, but whether KLRG1 is simply a convenient marker of cells with the appropriate phenotype or a facilitator in programming T_RM_ cells is unclear [27,94]. One study showed that KLRG1^low^ T cells accumulate in the gut resulting in T_RM_ cell formation [36]. T-bet negatively regulates CD8^+^ T_RM_ cells formation which has been shown to favor the development of CD103^+^ T_RM_ cells in the lungs in a dose-dependent manner [48]. In the present study, both CD103^+^ and CD103^-^ CD8^+^ T_RM_ cells induced by mucosal znBAZ immunization were shown to express low levels of CD127, KLRG1, and T-bet.

Anti-CD8 mAb treatment did not alter the expression patterns of CD103^+^ and CD103^-^ CD8^+^ T_RM_ cells in the lungs, but did slightly reduce their MFIs. Anti-CD8 mAb treatment cannot directly affect T_RM_ cells in the epithelium, but such treatment may reduce the replenishment of these CD8^+^ T_RM_ cells, and ultimately result in reduction of CD8^+^ T_RM_ cells in the lung parenchyma and airways.

The respiratory mucosa is a major site for invasion by pathogens including *Brucella*. Lung parenchyma and airways provide important immunological niches used to prevent pulmonary infections [47,61]. CD8^+^ T_RM_ cells play a crucial role in controlling pulmonary infections by their strategic distribution within distinct anatomical compartments of the lungs [50–53]. CXCR3 is required for appropriate differentiation and localization of T_RM_ cells to mucosal tissue such as the epithelium of lung, small intestine and skin [27,32,34,47,66]. Previous studies demonstrated that protective CXCR3^hi^ memory CD8^+^ T cells, induced by viral infection, occupied the lung airways [51,56]. Another study identified CXCR3^lo^ CD8^+^ T_RM_ cells to be the initial responders to viral infection, and this subset preferentially localizes to the lung alveolar and bronchiolar walls of the pulmonary interstitium [47]. Mucosal vaccination with *Mycobacterium bovis* Bacille Calmette-Guérin (BCG) induced protective T_RM_ cells localized to lung airways and parenchyma to protect against tuberculosis [91]. The current study demonstrates that mucosal znBAZ immunization induces CD103^+^ and CD103^-^ CD8^+^ T_RM_ cells expressing CXCR3^lo^ and CXCR3^hi^ phenotypes distributed in the lung parenchyma and airways, respectively. These CXCR3-expressing CD103^+^ and CD103^-^ CD8^+^ T_RM_ cells could not be depleted by anti-CD8 mAb treatment, and the remaining CD8^+^ T_RM_ cells conferred 2.8 logs of protection against pulmonary wt *B*. *abortus* challenge (Fig 4). Mucosally znBAZ-vaccinated CD8^-/-^ mice were not protected against virulent *B*. *abortus* challenge, showing that indeed the induction of CD8^+^ T_RM_ cells is essential for protection against pulmonary challenge. Additionally, few or no CXCR3^-^ CD8^+^ T cells were observed after anti-CD8 mAb treatment following znBAZ immunization. Although CD8^+^ T cells were effectively depleted from the spleens of znBAZ-vaccinated mice, the remaining T_RM_ cells may have been sufficient to prevent the systemic spread, e.g., brucellae colonization of the spleen. Collectively, these data show the importance of CXCR3-expressing CD103^+^ and CD103^-^ CD8^+^ T_RM_ cells in the protection against pulmonary wt *B*. *abortus* challenge in mucosally znBAZ-vaccinated mice.

Our results show that mucosal znBAZ vaccination induced polyfunctional CD4^+^ T cells which produce IFN-γ and TNF-α. However, functional analysis revealed that these CD4^+^ T cells are not essential to protect against wt challenge since their depletion did not impact vaccine efficacy. This result does not rule out the possibilty that CD4^+^ T cells are important in the formation of CD8^+^ T_RM_ cells and in localization of CD8^+^ T_RM_ cells into the lungs. A recent study showed that CD4^+^ T cells are important for the formation of functional lung-resident CD8^+^ T cells and for directing CD8^+^ T cells to the airway epithelium [48,95]. For this reason, investigation of whether CD4^+^ T cells help the formation and localization of pulmonary CD8^+^ T_RM_ cells in the *Brucella* vaccine model would be interesting. It would also be of interest to learn how CD4^+^ T_RM_ cells are induced subsequent vaccination to brucellosis to determine, if these could then, confer protection. Notably, the levels of polyfunctional CD8^+^ T_RM_ cells were significantly reduced in the lungs subsequent to wt challenge, implicating their importance for protection. Splenic polyfunctional CD8^+^ T cells were also reduced following challenge. These studies demonstrate that effective mucosal vaccination can result in the appropriate stimulation of memory T cell responses required for protection against pulmonary and systemic *Brucella* infection.

## Supporting information

S1 Fig Sequence of znBAZ vaccination does not significantly change its efficacy. Groups of BALB/c mice (n = 9/group) were dosed with sPBS; primed by the intranasal (IN) and boosted by the oral routes; or primed by the oral and boosted by the IN routes with 1x10^9^ CFUs/dose. Mice were primed on day 0, and boosted on day 28; 4 wks post-boost, mice were IN challenged with 5x10^4^ CFUs of virulent wt *B*. *abortus* 2308. The extent of wt brucellae colonization of the (Figure S-1A) spleen and (Figure S-1B) lungs were measured 4 wks post-challenge. Data are the mean ± SEM of tissue CFU levels. Analysis of variance with One-way ANOVA Tukey’s multiple comparisons test was performed; *P<0.05, versus sPBS-dosed mice.
(TIF)

S2 Fig znBAZ enhances LRLN CD8^+^ T cell responses. BALB/c mice were primed and boosted as described in Fig 1A. On days 42 and 56, LRLN T cells were evaluated by flow cytometry analysis. Additional groups of mice subjected to the same immunization were challenged with wt *B*. *abortus* 2308 (5×10^4^ CFUs) on day 56. Four weeks post-challenge, LRLNs were isolated to measure the CD4^+^ and CD8^+^ T cell levels (n = 12 mice per group, data from two independent experiments). The difference was determined when compared to sPBS-dosed mice (****P<0.0001, ***P<0.001, **P<0.01, *P<0.05), or compared to RB51-vaccinated mice (^++++^P<0.0001, ^+++^P<0.001, ^++^P<0.01, ^+^P<0.05). Analysis of variance with Two-way ANOVA Tukey’s multiple comparisons test was done.
(TIF)

S3 Fig Cytokine expression by splenic CD4+ and CD8^+^ T cells. BALB/c mice were primed and boosted with sPBS, RB51, and znBAZ as described in Fig 1A. At pre- and post-wt *B*. *abortus* 2308 challenge, mice were analyzed for the expression of proinflammatory cytokines by splenic (Figure S-3A) CD4^+^ and (Figure S-3B) CD8^+^ T cells (n = 12 mice per group, data from two independent experiments). The difference was determined when compared to sPBS-dosed mice (****P<0.0001, ***P<0.001, **P<0.01, *P<0.05), or compared to RB51-vaccinated mice (^++++^P<0.0001, ^+++^P<0.001, ^++^P<0.01, ^+^P<0.05).
(TIF)

S4 Fig In vivo depletion of T cells using anti-CD4 and anti-CD8 mAbs results in the loss of the respective splenic T cell subset. BALB/c mice were primed and boosted with sPBS, RB51, and znBAZ as described in Fig 1A. On day 56, all mice were challenged with wt *B*. *abortus* 2308, and on days 55 (one day before challenge), 57, 62, and 66, mice were IP treated with isotype control, anti-CD4, or anti-CD8α mAb. On day 70 (2 weeks after challenge), harvested spleens were analyzed for T cell profiles by total cell numbers (n = 12 mice per group, data from three independent experiments). The difference was determined when compared to Isotype Ab-dosed mice (****P<0.0001, ***P<0.001, **P<0.01, *P<0.05), or compared with anti-CD4 mAb-treated mice (^++++^P<0.0001, ^+++^P<0.001, ^++^P<0.01, ^+^P<0.05). Analysis of variance with Two-way ANOVA Tukey’s multiple comparisons test was performed.
(TIF)

S5 Fig Memory CD103^+^ CD69^+^ CD4^+^ T cells. BALB/c mice were primed and boosted with sPBS, RB51, and znBAZ as described in Fig 1A. At pre- or post-wt *B*. *abortus* 2308 challenge, lungs were analyzed for the expression of memory CD4^+^ T cell subsets on days 42 and 56 (pre-challenge), as well as on day 84 (post-challenge). Data depict n = 12 mice per group from three independent experiments. The difference was determined when compared to sPBS-dosed mice (****P<0.0001, ***P<0.001, **P<0.01, *P<0.05). Analysis of variance with Two-way ANOVA Tukey’s multiple comparisons test.
(TIF)

S6 Fig CD103^+^ and CD103^-^ CD8^+^ T_RM_ cells present in the lungs from znBAZ-vaccinated mice are CD44^+^, and not those in the lungs from PBS-dosed or RB51-vaccinated BALB/c mice. Depicted are the immunofluorescent results of staining using a polyclonal anti-CD44 Ab, showing that CD44^+^ is most apparent in the lungs from (C) znBAZ-vaccinated mice, but less evident in the lungs from (A) PBS-dosed or (B) RB51-vaccinated mice. Magnification is 400x; line represents 50 μm in length.
(TIF)

S7 Fig CXCR3 expression by CD103^+^ and CD103^-^CD8^+^T_RM_ cells in lung parenchyma and BALF. BALB/c mice were primed and boosted with sPBS or znBAZ as described in Fig 1A. On days 55, 57, 62, and 66, mice were IP treated with isotype or anti-CD8α mAb. On day 70, mice were evaluated for CXCR3 expression by CD103^+^ and CD103^-^CD8^+^ T_RM_ cells in lung (A) parenchyma and (B) BALF ± anti-CD8 mAb treatment. Representative data depict n = 12 mice per group from three independent experiments.
(TIF)

S8 Fig Lung resident (Resid) versus recirculating (Recir) CD8^+^T cells. BALB/c mice were primed and boosted with sPBS or znBAZ as described in Fig 1A. On days 55, 57, 62, and 66, mice were IP treated with isotype or anti-CD8α mAb. On day 70, mice were *in vivo* labeled with anti-CD8 mAb by IV injection, and mice were euthanized 10 mins later. Lung parenchyma and BALF cells were collected to evaluate their resident and recirculating CD8^+^T cell profiles in lung parenchyma and airways ± anti-CD8 mAb treatment. Representative data depict n = 12 mice per group from three independent experiments.
(TIF)
