# Supplementary figures and images for: Targeting resident memory T cell immunity culminates in pulmonary and systemic protection against Brucella infection
Source: PLoS Pathog. 2020 Jan 17;16(1):e1008176. doi: 10.1371/journal.ppat.1008176 (PMC6968852; doi:10.1371/journal.ppat.1008176)

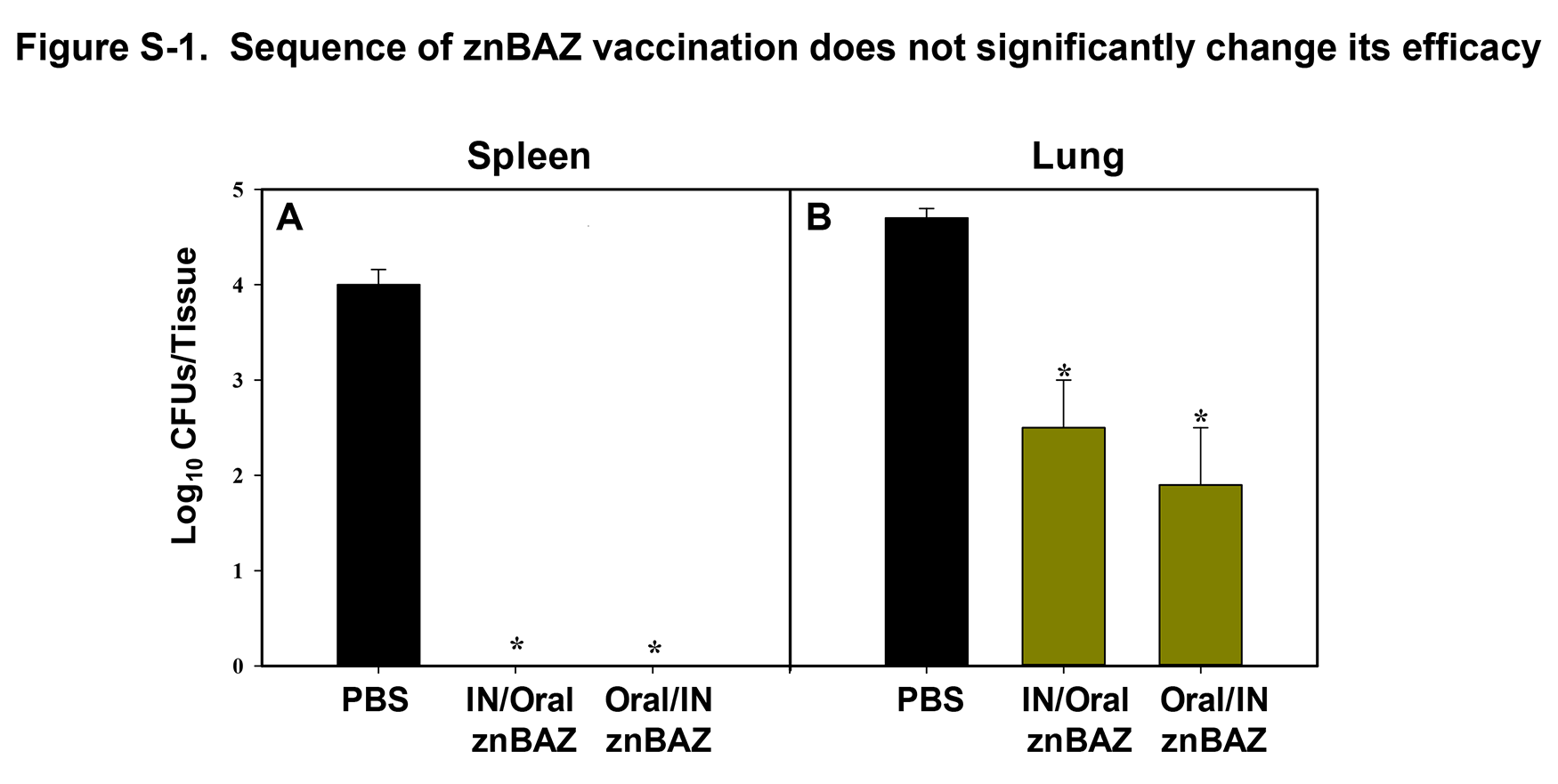

Supplement: S1 Fig — Groups of BALB/c mice (n = 9/group) were dosed with sPBS; primed by the intranasal (IN) and boosted by the oral routes; or primed by the oral and boosted by the IN routes with 1x10^9^ CFUs/dose. Mice were primed on day 0, and boosted on day 28; 4 wks post-boost, mice were IN challenged with 5x10^4^ CFUs of virulent wt B. abortus 2308. The extent of wt brucellae colonization of the (Figure S-1A) spleen and (Figure S-1B) lungs were measured 4 wks post-challenge. Data are the mean ± SEM of tissue CFU levels. Analysis of variance with One-way ANOVA Tukey’s multiple comparisons test was performed; *P<0.05, versus sPBS-dosed mice. (TIF) [file ppat.1008176.s001.tif]

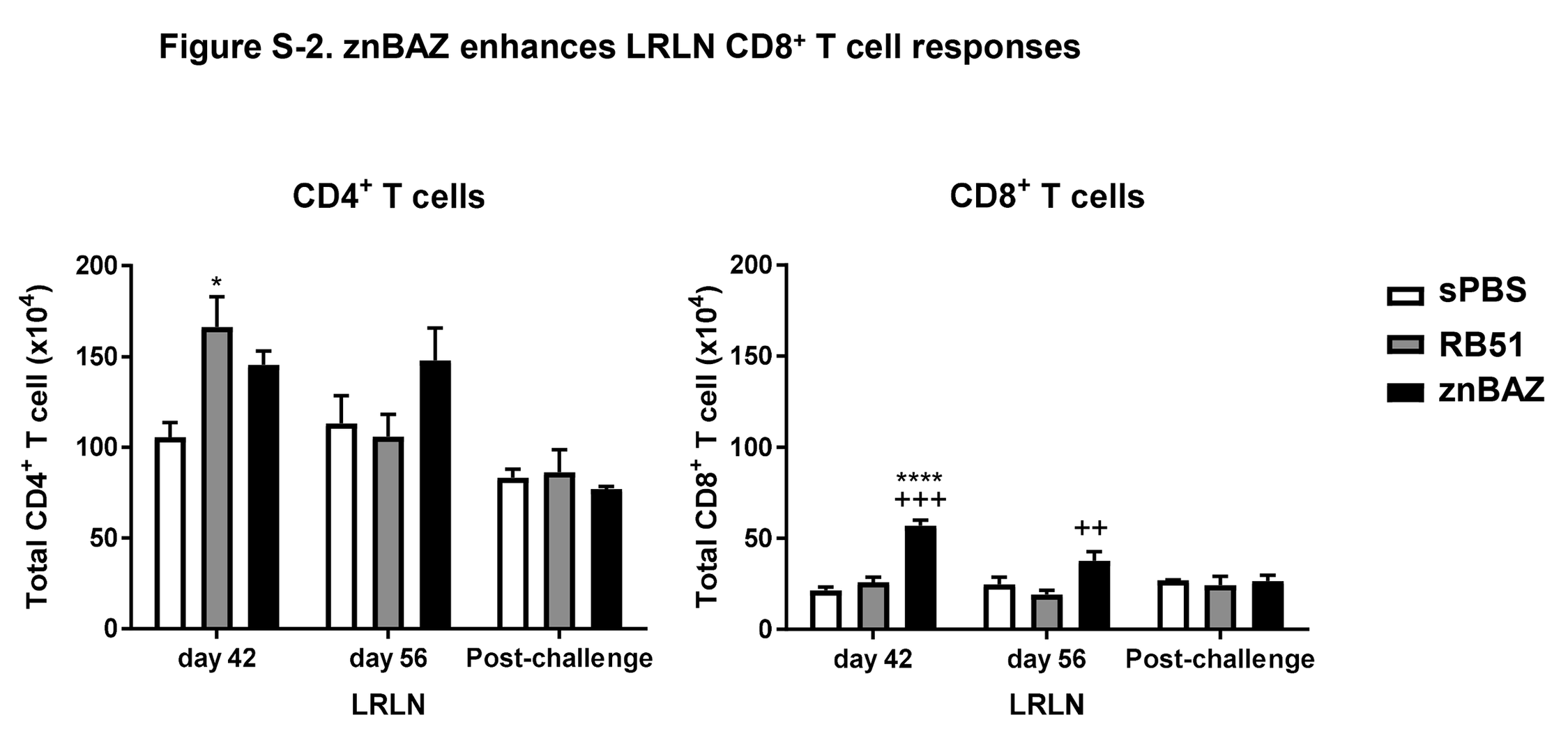

Supplement: S2 Fig — BALB/c mice were primed and boosted as described in Fig 1A. On days 42 and 56, LRLN T cells were evaluated by flow cytometry analysis. Additional groups of mice subjected to the same immunization were challenged with wt B. abortus 2308 (5×10^4^ CFUs) on day 56. Four weeks post-challenge, LRLNs were isolated to measure the CD4+ and CD8+ T cell levels (n = 12 mice per group, data from two independent experiments). The difference was determined when compared to sPBS-dosed mice (****P<0.0001, ***P<0.001, **P<0.01, *P<0.05), or compared to RB51-vaccinated mice (++++P<0.0001, +++P<0.001, ++P<0.01, +P<0.05). Analysis of variance with Two-way ANOVA Tukey’s multiple comparisons test was done. (TIF) [file ppat.1008176.s002.tif]

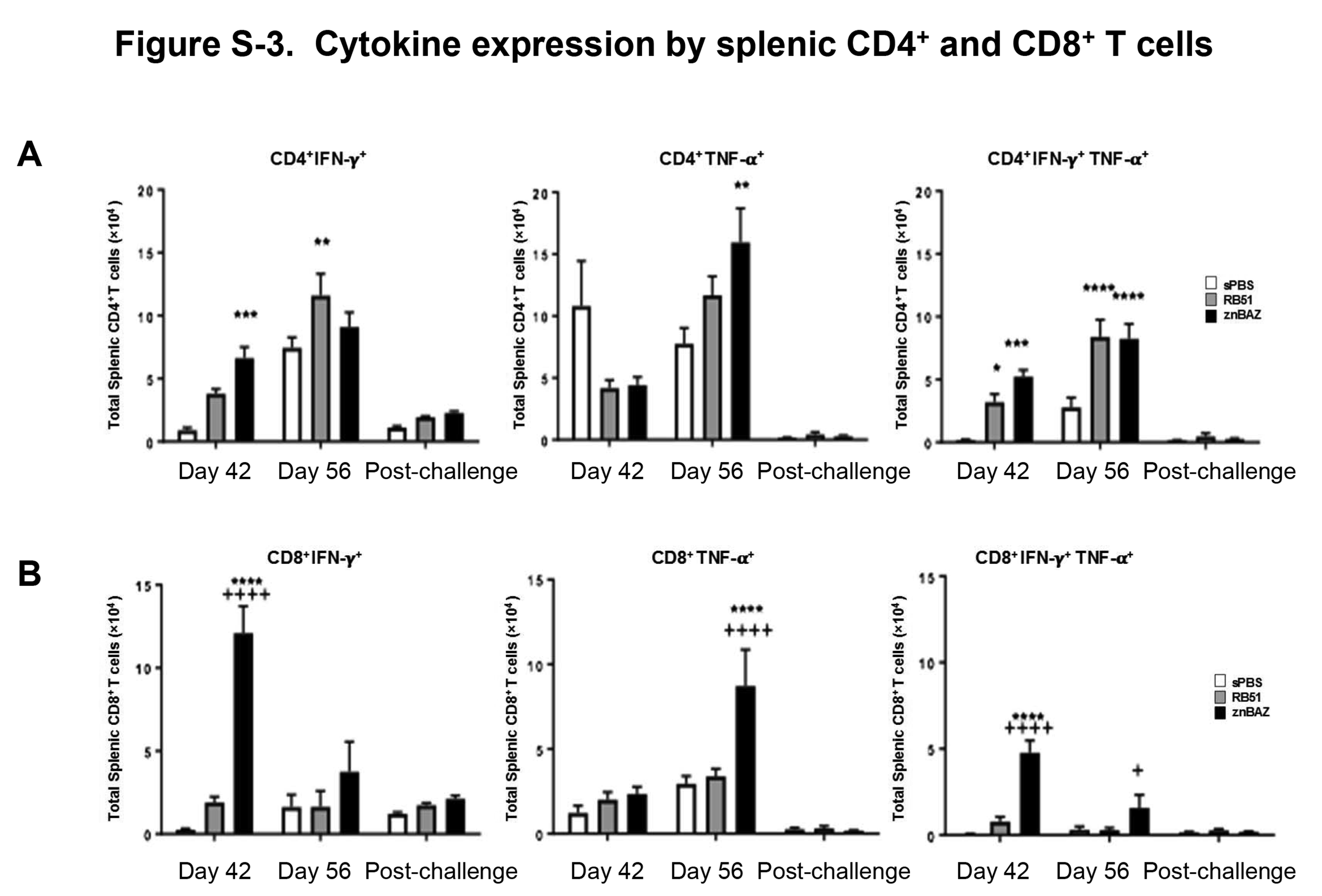

Supplement: S3 Fig — BALB/c mice were primed and boosted with sPBS, RB51, and znBAZ as described in Fig 1A. At pre- and post-wt B. abortus 2308 challenge, mice were analyzed for the expression of proinflammatory cytokines by splenic (Figure S-3A) CD4+ and (Figure S-3B) CD8+ T cells (n = 12 mice per group, data from two independent experiments). The difference was determined when compared to sPBS-dosed mice (****P<0.0001, ***P<0.001, **P<0.01, *P<0.05), or compared to RB51-vaccinated mice (++++P<0.0001, +++P<0.001, ++P<0.01, +P<0.05). (TIF) [file ppat.1008176.s003.tif]

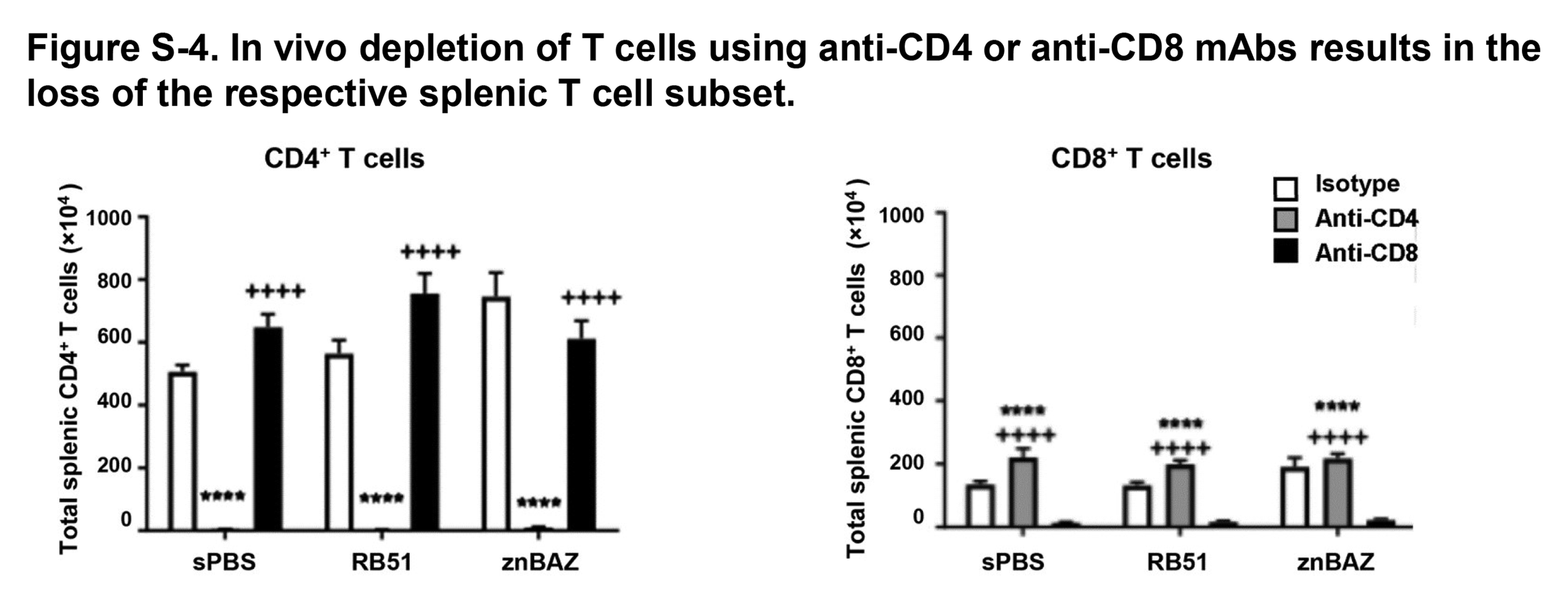

Supplement: S4 Fig — BALB/c mice were primed and boosted with sPBS, RB51, and znBAZ as described in Fig 1A. On day 56, all mice were challenged with wt B. abortus 2308, and on days 55 (one day before challenge), 57, 62, and 66, mice were IP treated with isotype control, anti-CD4, or anti-CD8α mAb. On day 70 (2 weeks after challenge), harvested spleens were analyzed for T cell profiles by total cell numbers (n = 12 mice per group, data from three independent experiments). The difference was determined when compared to Isotype Ab-dosed mice (****P<0.0001, ***P<0.001, **P<0.01, *P<0.05), or compared with anti-CD4 mAb-treated mice (++++P<0.0001, +++P<0.001, ++P<0.01, +P<0.05). Analysis of variance with Two-way ANOVA Tukey’s multiple comparisons test was performed. (TIF) [file ppat.1008176.s004.tif]

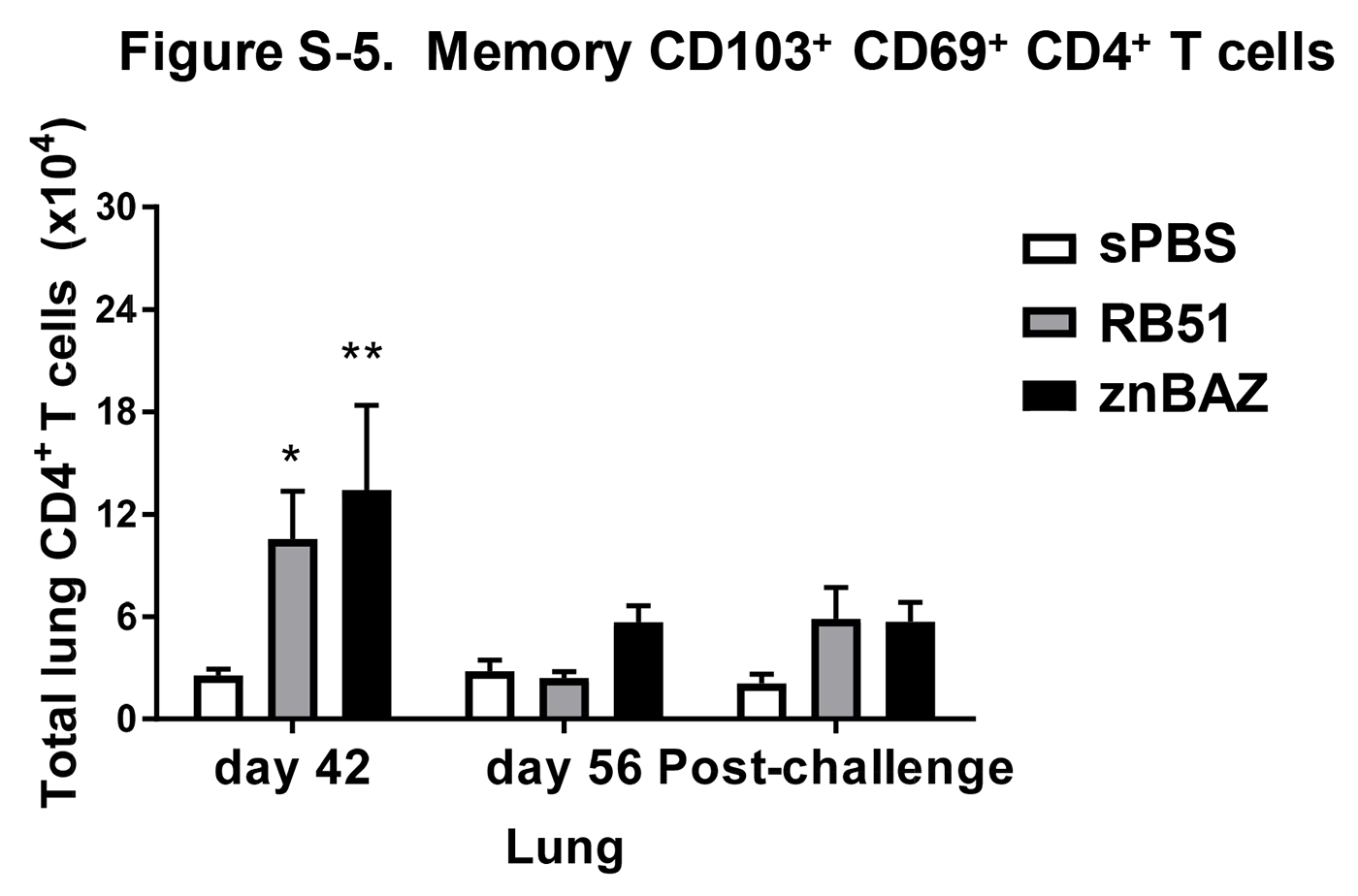

Supplement: S5 Fig — BALB/c mice were primed and boosted with sPBS, RB51, and znBAZ as described in Fig 1A. At pre- or post-wt B. abortus 2308 challenge, lungs were analyzed for the expression of memory CD4+ T cell subsets on days 42 and 56 (pre-challenge), as well as on day 84 (post-challenge). Data depict n = 12 mice per group from three independent experiments. The difference was determined when compared to sPBS-dosed mice (****P<0.0001, ***P<0.001, **P<0.01, *P<0.05). Analysis of variance with Two-way ANOVA Tukey’s multiple comparisons test. (TIF) [file ppat.1008176.s005.tif]

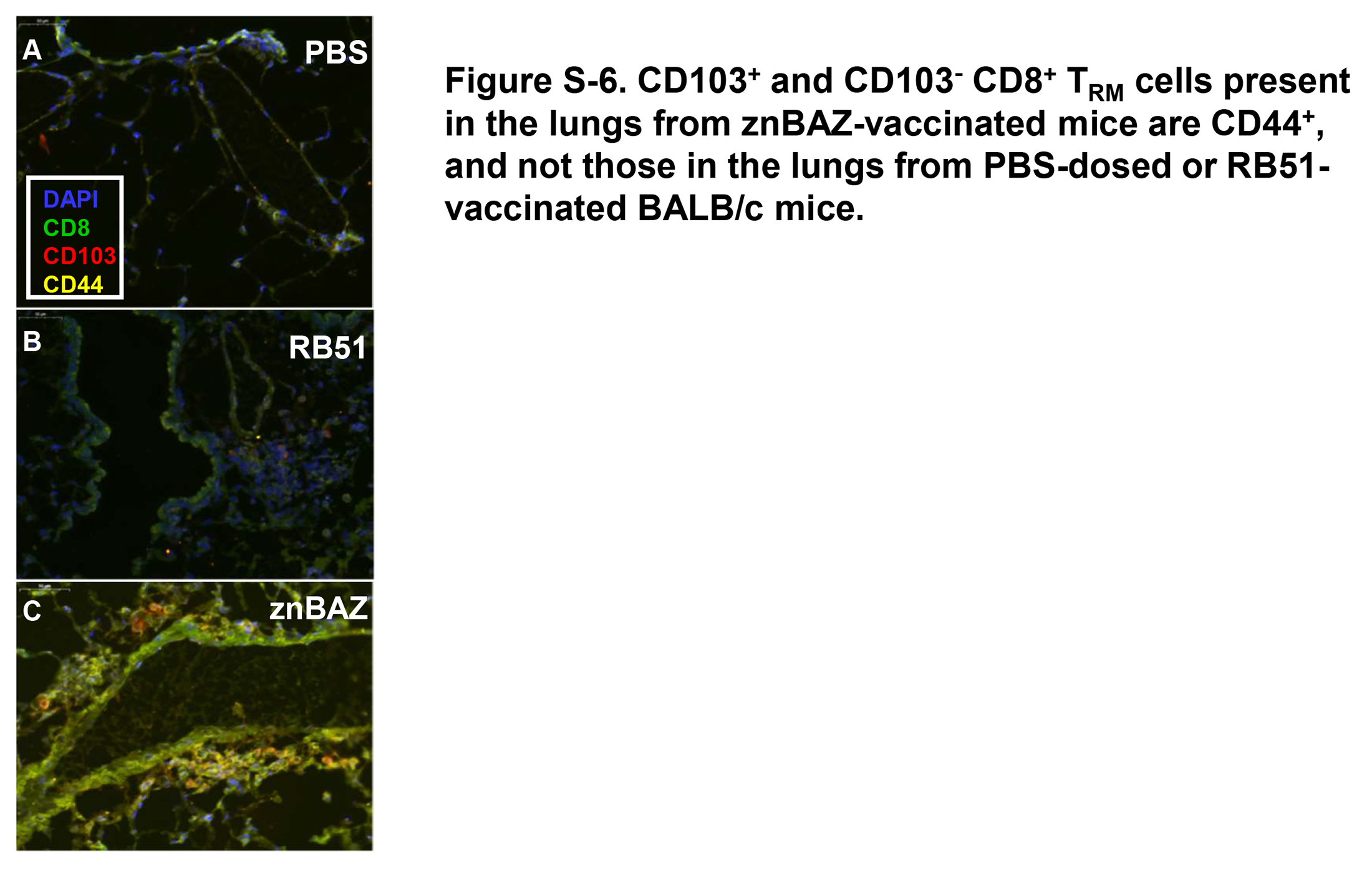

Supplement: S6 Fig — Depicted are the immunofluorescent results of staining using a polyclonal anti-CD44 Ab, showing that CD44+ is most apparent in the lungs from (C) znBAZ-vaccinated mice, but less evident in the lungs from (A) PBS-dosed or (B) RB51-vaccinated mice. Magnification is 400x; line represents 50 μm in length. (TIF) [file ppat.1008176.s006.tif]

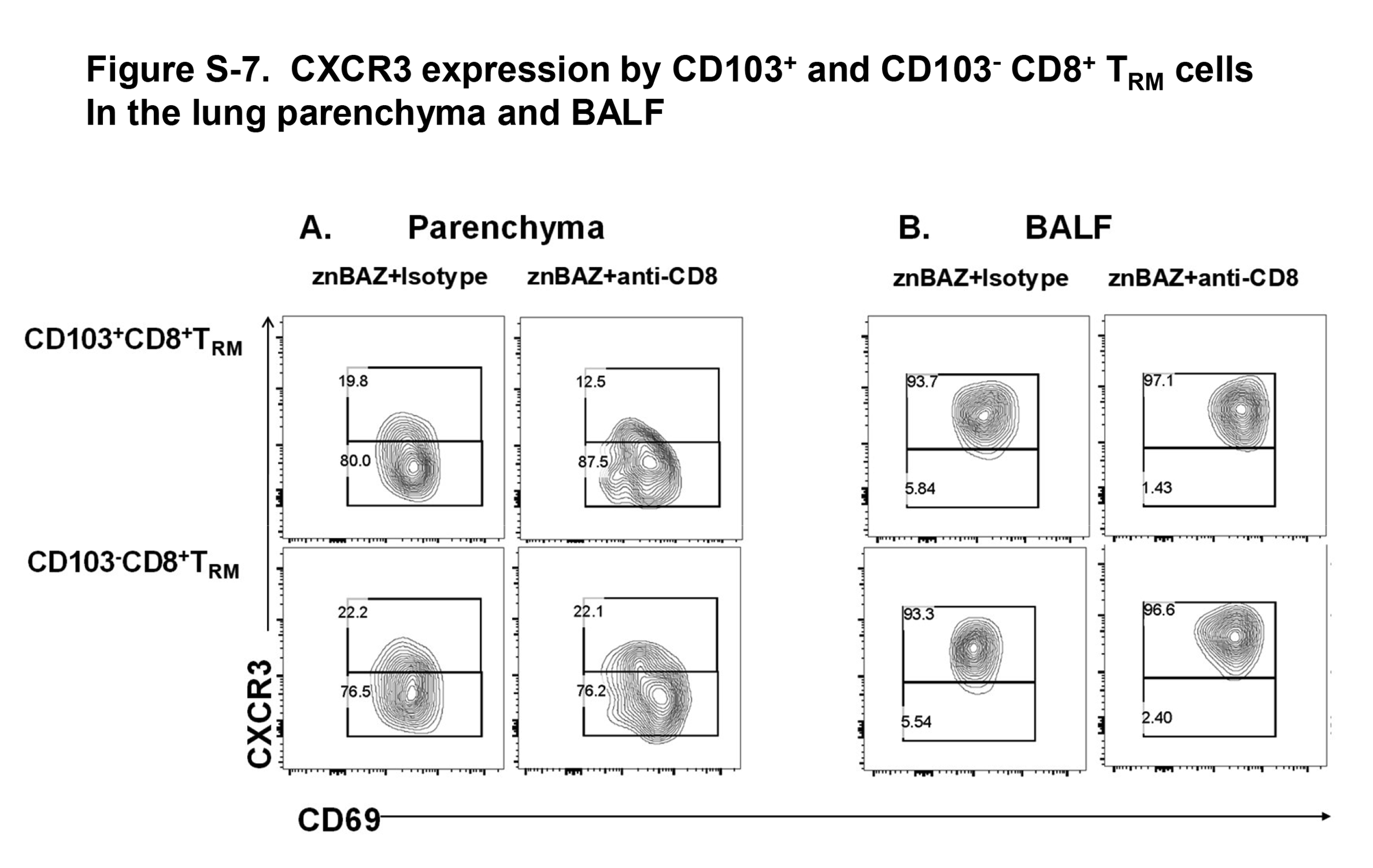

Supplement: S7 Fig — BALB/c mice were primed and boosted with sPBS or znBAZ as described in Fig 1A. On days 55, 57, 62, and 66, mice were IP treated with isotype or anti-CD8α mAb. On day 70, mice were evaluated for CXCR3 expression by CD103+ and CD103-CD8+ TRM cells in lung (A) parenchyma and (B) BALF ± anti-CD8 mAb treatment. Representative data depict n = 12 mice per group from three independent experiments. (TIF) [file ppat.1008176.s007.tif]

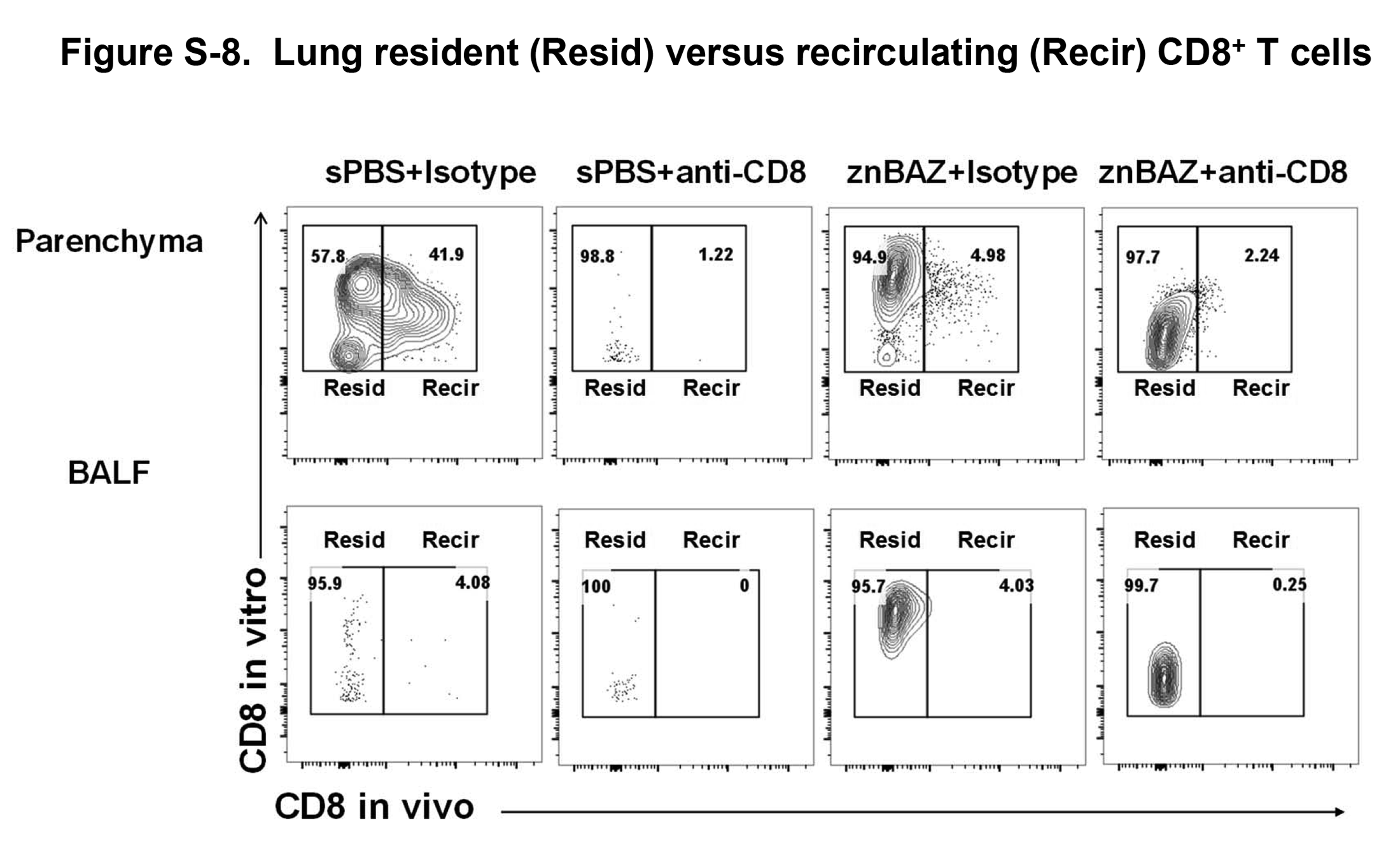

Supplement: S8 Fig — BALB/c mice were primed and boosted with sPBS or znBAZ as described in Fig 1A. On days 55, 57, 62, and 66, mice were IP treated with isotype or anti-CD8α mAb. On day 70, mice were in vivo labeled with anti-CD8 mAb by IV injection, and mice were euthanized 10 mins later. Lung parenchyma and BALF cells were collected to evaluate their resident and recirculating CD8+T cell profiles in lung parenchyma and airways ± anti-CD8 mAb treatment. Representative data depict n = 12 mice per group from three independent experiments. (TIF) [file ppat.1008176.s008.tif]
